# Transcriptional analysis of *C. elegans fmo*s at different life stages and their roles in ageing

**DOI:** 10.1007/s00438-024-02201-x

**Published:** 2024-12-05

**Authors:** Mohamed Said, Bill T. Ferrara, Andreea Aprodu, Filipe Cabreiro, Elinor P. Thompson, Jeremy Everett

**Affiliations:** 1 Faculty of Engineering and Science, University of Greenwich, Chatham Maritime, Kent, ME4 4TB UK; 2 Institute of Clinical Sciences, Imperial College London, London, W12 0NN UK; 3 Cologne Excellence Cluster for Cellular Stress Responses in Aging-Associated Diseases (CECAD), University of Cologne, Joseph Stelzmann Strasse 26, 50931 Cologne, Germany; 4 Present Address: Faculty of Pharmacy, October University for Modern Sciences and Arts, 6th October City, Egypt

**Keywords:** *C. elegans* worms, Gene mutation, Flavin-containing monooxygenases (FMOs), Transcription, Lifespan

## Abstract

Flavin-containing monooxygenases (FMOs) are present in most organisms including plants, fungi, bacteria, invertebrates and vertebrates, where they catalyse the oxidative metabolism of a range of xenobiotics and endogenous metabolites. FMOs have been associated with ageing and longevity in the mouse and in *C. elegans*. As all five FMOs of *C. elegans* share an evolutionary root with mouse and human FMO5, it was of interest to discover if effects on ageing and longevity persisted across the whole group. We therefore investigated the impact of *fmo* gene knockout (KO) in *C. elegans*. We found that *fmo-1*, *fmo-3* and *fmo-4* KO significantly extended *C. elegans* lifespan relative to wild type and, as previously reported, FMO-2 over-expression did likewise. The transcription levels of *C. elegans fmo* genes were determined throughout the life cycle (embryo, larva and adult) in wild type and in each mutant to discover if their expression was related to stages in ageing, and expression levels were compared to those in human and mouse. In wild type worms, *fmo-1* and *fmo-4* were the mostly highly transcribed genes (especially at the larval stage), whereas *fmo-2* and *fmo-3* were the least transcribed, at all stages. Notably, the knockout of *fmo-4* led to a 17- to 30-fold up-regulation of *fmo-2,* along with significantly increased levels of the other *fmo*s. This parallels recent findings in the long-lived *C. elegans tald-1* mutant where *fmo-2* was also significantly up-regulated and reinforces its importance in lifespan extension.

**Supplementary Information:**

The online version contains supplementary material available at 10.1007/s00438-024-02201-x.

## Introduction

The nematode *C. elegans* is one of the most utilised biological research models in different fields such as genomics, neuroscience, cell biology and ageing, because of its short life cycle (ca. 3 days under optimal growth conditions), genetic homology with humans and the ease of its practical manipulation in the laboratory (Altun and Hall 2009; Corsi et al. 2015). FMOs are important in the metabolism of drugs, dietary-derived compounds, pesticides, and of endogenous substrates containing nucleophilic heteroatoms, mainly sulphur, nitrogen and phosphorous. FMOs oxygenate their substrates via binding to an FAD prosthetic group and interacting with an NADPH cofactor (Phillips and Shephard 2008, 2017). Recently it has become clear that FMOs also have important roles in endogenous metabolism in mammals. For example, *fmo5* knockout (KO) mice exhibit an age-related phenotype with low body fat and weight loss despite higher food intake, and they have lower blood glucose and cholesterol, and slowed metabolic ageing through pleiotropic effects (Malagon et al. 2015). More recently, it was shown in mice and humans that FMO1 catalyses the conversion of hypotaurine to taurine, utilising either NADPH or NADH as co-factor, solving a mystery of greater than 50 years as to the origin of this important amino acid in mammals (Veeravalli et al. 2020). Finally, hepatic FMO3 is the primary FMO responsible for trimethylamine *N*-oxide (TMAO) production from trimethylamine, so that genetic mutations affecting the production or activity of FMO3 result in the disorder trimethylaminuria (or fish-odour syndrome) (Dolphin et al. 1997; Yamazaki and Shimizu 2007; Shephard et al. 2012; Phillips and Shephard 2020). It is estimated that FMOs also play an important role in the global carbon and nitrogen cycles because of the number of surface ocean bacteria containing an FMO that functions as a trimethylamine monooxygenase (Chen et al. 2011).

The FMO protein family is highly conserved both genetically and structurally across all organisms from bacteria to humans, although not present in Archaea (Ziegler 2002; Krueger and Williams 2005; Chen et al. 2011). The human genome possesses 11 *FMO* genes, five of which are functional, *FMO1-5*, with *FMO6P*–*FMO11P* being predicted pseudogenes (Hernandez et al. 2004; Phillips and Shephard 2017). In humans, *FMO1-4* and *FMO6P* are located on chromosome 1, in the region q24.3, whereas *FMO5* is located ~ 26 Mb closer to the centromere, at 1q21.1. Moreover, there is another cluster containing pseudogenes *FMO7P* to *FMO11P* located in the region 1q24.2 (Hernandez et al. 2004; Phillips and Shephard 2017). The mouse genome possesses five functional *Fmo* genes: *Fmo1-5*, which are the orthologues of the corresponding human genes. Four additional genes: *Fmo6, Fmo9, Fmo12* and *Fmo13* are predicted to be functional but the capabilities of their protein products are unknown (Phillips and Shephard 2017). Mouse *Fmo5* is located separately from the rest of *Fmo*s (*Fmo1-4),* as seen in the human genome, but on chromosome 3.

The *C. elegans* genome also contains five functional *fmo* genes: *fmo-1* and *fmo-2* are clustered together on chromosome 4, but *fmo-4* and *fmo-5* are located on chromosome 5 and *fmo-3* is located on chromosome 3 (Petalcorin et al. 2005) (Figure S1). *C. elegans* lacking *fmo-1, -4*, and* -5* have neurodevelopmental defects including growth cones with excessive (longer) filopodial protrusions compared with wild type, whereas FMO-5 transgenic expression inhibits growth cone protrusion (Gujar et al. 2017). FMOs have a role in inhibition of growth cone protrusion downstream of UNC-6/ Netrin signalling by possibly promoting phosphorylation of UNC-33/CRMP or by directly oxidising F-actin (Gujar et al. 2017).

In nematodes and mammals, hypoxia-inducible factor (HIF) proteins have a central role in responding to environmental oxygen changes (Jiang et al. 2001). Stabilization of HIF in mammals through loss of the E3 ubiquitin ligase von Hippel-Lindau (VHL) protein led to a disease characterized by renal carcinomas (Ivan and Kaelin 2001), whereas in *C. elegans*, loss of *vhl-1*, improved proteostasis and increased lifespan (Leiser et al. 2015). Following a screen of genes downstream of *vhl-1* mutants in *C. elegans* to investigate how hypoxic signalling slowed ageing in *C. elegans, fmo-2* was identified as necessary for the associated longevity and health phenotypes of HIF-1 response (Leiser et al. 2015). Intestinal FMO-2 was also upregulated by dietary restriction (DR) and was needed for DR-mediated lifespan extension (Uno and Nishida 2016). Intestinal overexpression (OE) of FMO-2 was also sufficient to confer these benefits on its own and increased lifespan, improved healthy lifespan and enhanced proteostasis in worms (Leiser et al. 2015). Further, intestinal transcription of *fmo-2* was regulated through serotonergic signalling originating in neurons, subsequently activating the transcription factor HLH-30 in the intestine (Leiser et al. 2015). FMO-2 is thus an enzyme both necessary and sufficient for a majority of the beneficial effects of either of these longevity pathways (Leiser et al. 2015).

The association of FMOs with metabolic ageing in different species (Rossner et al. 2017) raised the question as to whether *fmo-2* is the only *fmo* involved, or whether other *fmo*s have a role in ageing in *C. elegans*? Answering this question might aid understanding of the evolution and relevance of FMO function both in and beyond this useful model. We addressed this by evaluating: (i) the impact of systematic knockout of *C. elegans fmo* genes on longevity; (ii) whether transcription levels of *fmo* genes indicated roles at particular points in the lifespan, and (iii) if transcription and phenotypes in *fmo* mutant worms suggested redundancy at important life stages that would support bioinformatics predictions that the *C. elegans* genes are all the result of ancestral FMO5 duplication.

## Results

### Sequence alignment of *C. elegans*, mouse and human FMOs

**Fig. 1:**
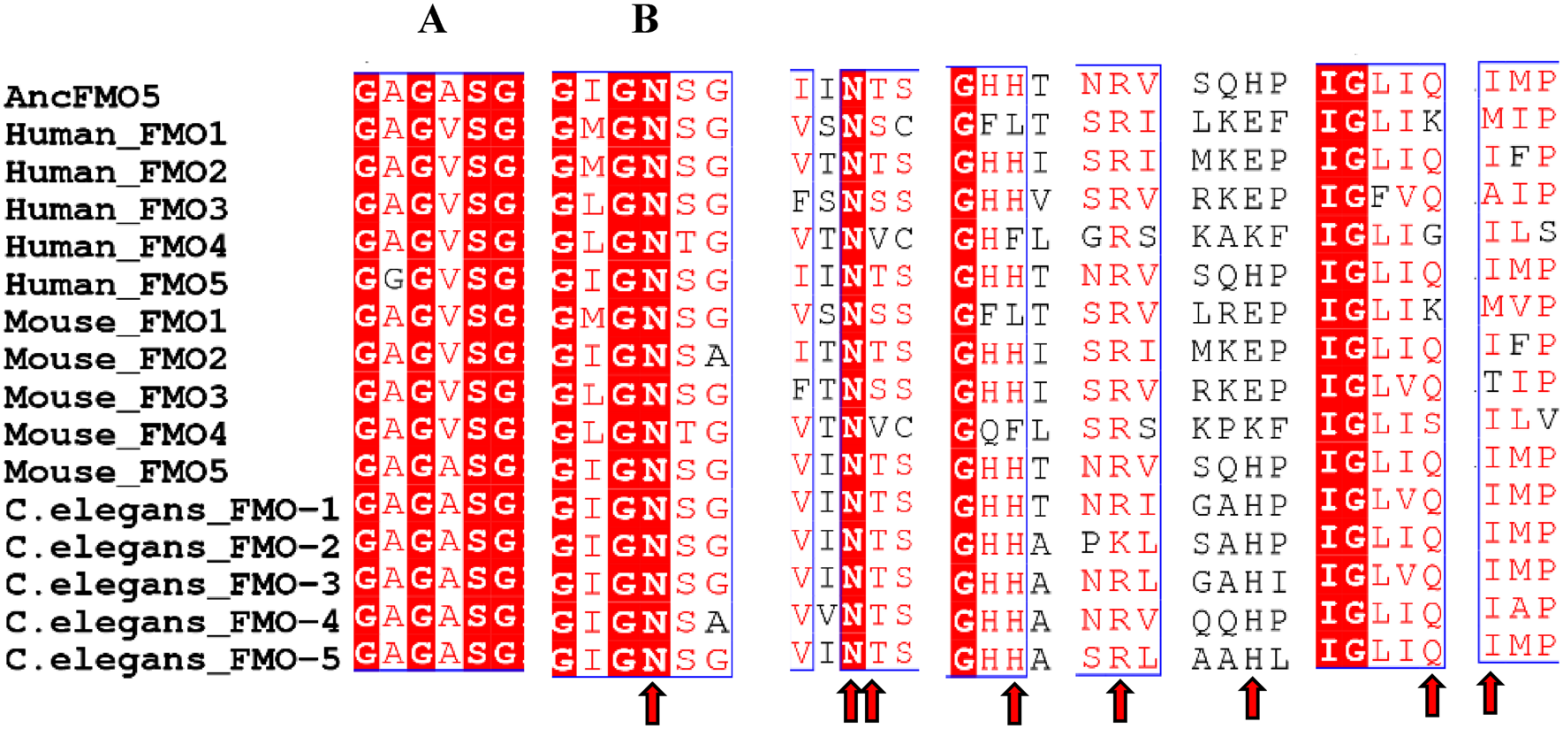
Amino acid sequence alignment of FMOs across species indicating the regions containing essential FAD (**A**) and NADP binding (**B**) domains and the eight essential residues (red arrowheads) in the catalytic active site among reconstructed ancestral (Anc) mammalian FMOs. Alignment Figures were generated using Clustal Omega (Sievers et al. 2011) and ESPript 3.0 (Robert and Gouet 2014)

FAD and NADPH binding motifs in FMOs were identified as GxGxxG and GxGxxG/A respectively in mouse (Phillips and Shephard 2017). Moreover, eight amino acids (Asn 62, Thr 63, His 151, Asn 195, Arg 224, His 282, Gln 373 and Ile 378) are essential residues for the catalytic active site in ancestral mammalian FMOs (Nicoll et al. 2020). Alignment of the amino acid sequences of the FMOs of *C. elegans*, *H. sapiens* and *M. musculus* using ancient mammalian FMOs (Nicoll et al. 2020) showed high conservation in the essential motifs and revealed the putative catalytic residues (Fig. 1; Figure S2).

### Transcription levels of *fmo* genes through *C. elegans* life cycle

**Fig. 2:**
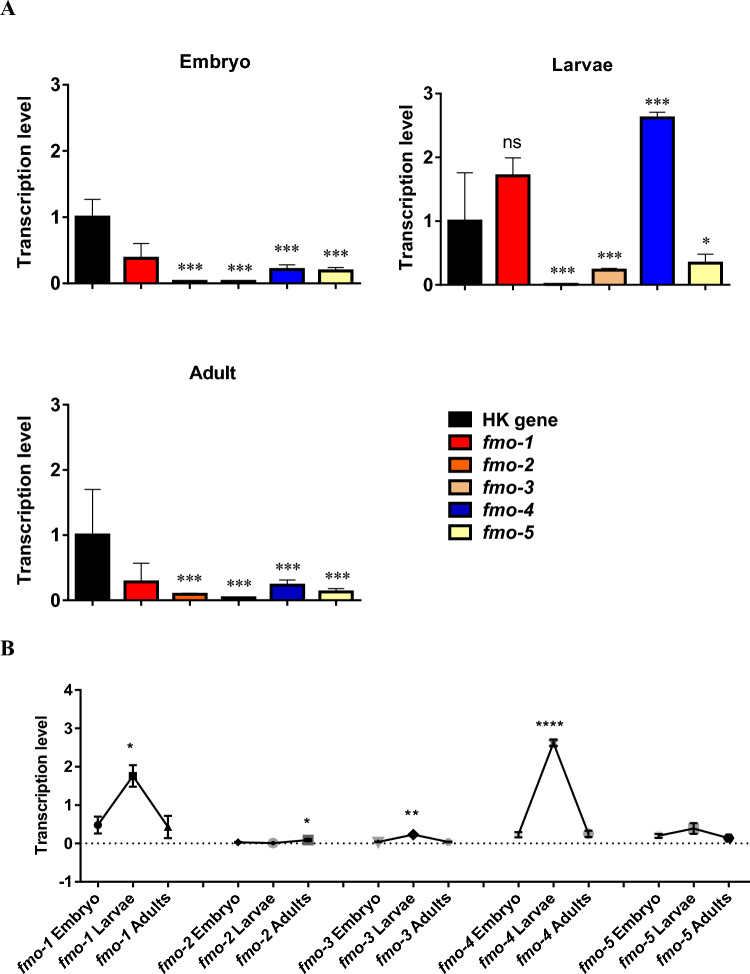
**A** Transcriptional analysis of the five *fmo* genes (*fmo1-5*) in embryo, larvae (L2; day 1 post hatching) and adult wild type *C. elegans* using qRT-PCR. Mean (± SEM) analysed from three biological repeats, normalised to the HK genes *pmp-3* and F35G12.2. HK, housekeeping genes. Each analysis was run in triplicate. One-way ANOVA using post hoc Dunnett’s test that compares each *fmo* gene at each stage to that of HK gene. **B** Transcription levels of the five *fmo* genes (*fmo1-5*). Mean (± SEM) analysed from three biological repeats, normalised to the HK genes *pmp-3* and F35G12.2. HK, housekeeping genes. Each analysis was run in triplicate. One-way ANOVA using post hoc Tukey’s test; ns, *P* > 0.05; *, *P* < 0.05; **, *P* < 0.01; ***, *P* < 0.001; **** *P* < 0.0001

The transcription levels of each *fmo* gene at embryo, larvae (L2; day 1 post hatching), and adult life stages were analysed (Fig. 2). *fmo-1* was the most highly transcribed at embryo and adult stages, whereas *fmo-2* and *fmo-3* were the least transcribed genes at all three stages (Fig. 2). Interestingly, *fmo-1* and *fmo-4* transcription was highly upregulated at larvae stage compared to the rest of *fmo*s.

The transcriptional patterns of *fmo-1, -3, -4* and* -5* of *C. elegans* through development were almost the same. All began with lower transcription level at embryo stage, reaching the peak of their transcription at larvae stage and then decreasing to the adult stage. In contrast, the transcriptional pattern of *fmo-2* was different, as its highest transcription level was at adult stage, and lowest at larvae stage (Fig. 2; Figure S3).

### The effect of *fmo* knockout on homologue transcription

Because of the upregulation of *fmo-1* and *fmo-4* at the larvae stage (L2; day 1 post hatching), we investigated the possible redundancy or shared roles in *C. elegans fmos* by comparing transcription levels in wild type versus *fmo-1* KO and *fmo-4* KO at the same stage. All *fmo* genes (*fmo1-3* and -*5*) were up-regulated significantly in *fmo-4* KO and their fold changes were 1.8, 30, 4.8 and 3.8 respectively, with the upregulation of *fmo-2* being a remarkable 30-fold (Figure S4A). Repeat measurement of this uniquely large change showed a fold-change of ca 17 (*p* < 0.001, Figure S5) in good agreement. In addition, no significant change was observed in *hlh-30* expression in the *fmo-*4 KO worm (Figure S5). By contrast, *fmo-2, -3 and-5* were the only up-regulated genes in the case of *fmo-1* KO with fold changes of 3.6, 2.3 and 3.3 respectively (Figure S4B).

Given the reported association between *C. elegans fmo-2* upregulation and increased lifespan (Petalcorin et al. 2005), transcription levels of *fmo* genes were also determined for the larvae (L2; day 1 post hatching; the stage with highest *fmo* levels in wild type worm) for the *fmo-2* KO and *fmo-2* OE mutants. *fmo-4* was down-regulated significantly in *fmo-2* OE (threefold; *P* = 0.0001), and surprisingly, in *fmo-2* KO as well (threefold; *P* value; *P* = 0.0025), whereas the remaining *fmo*s (*fmo1-3* and *fmo-5*) showed no significant changes. The results indicated that there is a somewhat complex link between *C. elegans fmo-2* and *fmo-4* and they may share similar functions (Figure S4C-D).

No significant changes in the transcription of any *fmo* was found on knockout of *fmo-3* (Figure S4E and S6). In the case of knockout of *fmo-5*, the only significant change was a ca 3.5-fold increase in the expression of *fmo-2* (Figure S4F and S7).

### Disruption of *C. elegans fmo* genes can extend lifespan

**Fig. 3:**
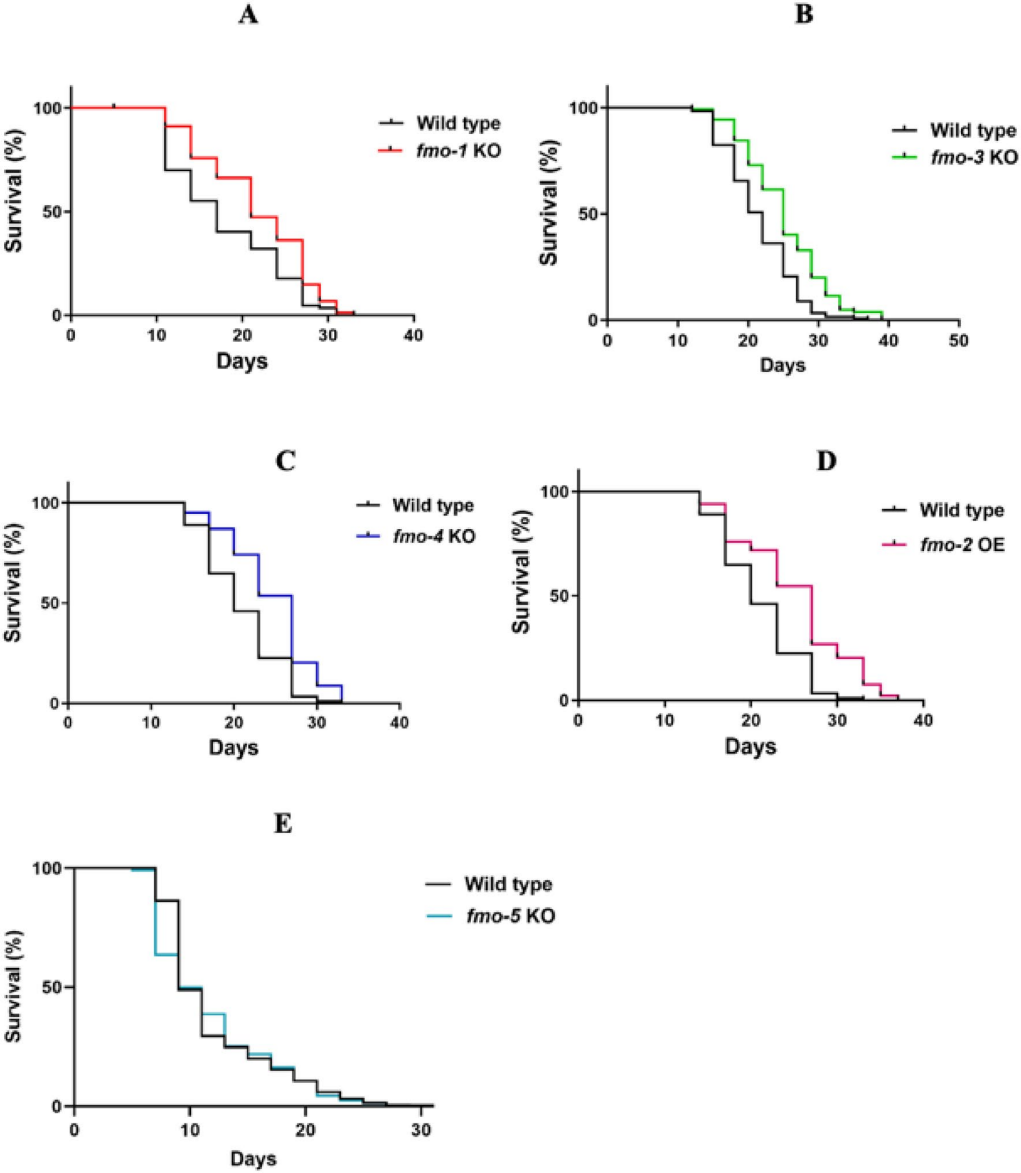
Kaplan–Meier survival curves and corresponding *p* values using log rank test for: **A** Wild type and fmo-1 KO worms, ***, *p* < 0.001; **B** Wild type and fmo-3 KO worms, ****, *p* < 0.0001; **C** Wild type and fmo-4 KO worms, ****, *p* < 0.0001; **D** Wild type and fmo-2 OE mutants, ****, *p* < 0.0001; **E** Wild type and fmo-5 KO worms, NS, *p* = 0.28

Based on the role of mouse *FMO5* in slowing metabolic ageing (Malagon et al. 2015) and of *C. elegans fmo-2* in healthspan and longevity (Leiser et al. 2015), we hypothesised, based on their sequence similarity, that other *C. elegans FMOs* might regulate or influence lifespan*.* Longevity assays of wild type worms versus the following *fmo* mutations: (*fmo-1* KO [*fmo-1*(*ok405*) *IV*]*, fmo-2* KO [*fmo-2*(*ok2147*) *IV*], *fmo-3* KO [*fmo-3(ok354) III*], *fmo-4* KO [*fmo-4*(*ok294*) *V*], *fmo-5* KO (tm2438) and FMO-2 OE [*seaSi40 I; unc-119(ed3) III*] were conducted to investigate changes in worm lifespan. Knocking out *fmo-1*, *-3*, *-4* and overexpression of *fmo-2* extended *C. elegans* lifespan relative to wild type whereas *fmo-5 KO* (Fig. 3) and *fmo-2* KO (Figure S8) had no effect on lifespan.

## Discussion

Alignment of *C. elegans*, mouse and human using ancient mammalian FMOs (Nicoll et al. 2020) illustrates their highly conserved catalytic residues (Fig. 1; Figure S2). A recent preprint reported that *C. elegans* FMO-2 and mammalian FMO5 possess highly conserved catalytic residues and the overall identity of murine FMO5 and *C. elegans* FMO-2 was approximately 43%, Choi et al. (2021) agreeing with our alignment results here. This indicates that the enzymatic activity and function of *C. elegans* and mammalian FMOs could be conserved.

Previous work has shown that knockout of the Fmo5 gene can have significant impact upon metabolic ageing, fat metabolism and other metabolic processes in mice (Malagon et al. 2015; Veeravalli et al. 2022). This raised the question as to what effect different fmo gene mutants would have on *C. elegans* lifespan. In this study we analysed fmo transcription levels at three key stages of the life cycle in wildtype *C. elegans*. We also studied the impact of the knockout of each of the five fmos by performing systematic survival assays for each mutant and assessing likely functional redundancy through *fmo* transcriptional analysis in the mutants.

In wildtype *C. elegans fmos,* transcriptional analysis showed that the pattern of *fmo-2* transcription was different from that of other *fmo*s, with highest transcription at adult stage, whereas transcription of *fmo-1, -3, -4* and *-5* was highest at larvae stage. A recent study reported that in different FMO-OE cell lines, stress resistance was improved, Huang et al. (2021) as already seen in *fmo-2* OE worms (Leiser et al. 2015). Moreover, *C. elegans* FMO-2 and mammalian FMO5 had similar oxidative activity toward tryptophan, suggesting that they may perform similar metabolic roles in both species (Choi et al. 2021).

Interestingly at the larvae stage, the transcription of *fmo-1* and *fmo-4* was increased by ca 3.5- and 10.8-fold, respectively relative to their transcription at the embryo stage. This indicates their importance in the early life cycle, and possible roles in the development of *C. elegans*. In fact, *fmo-4* and *fmo-1* were the most highly transcribed *fmo* genes at all three key life cycle stages, whereas *fmo-2* and *fmo-3* were the least transcribed *fmo* genes in wild type *C. elegans*. A study on *C. elegans* using transcriptional GFP or β-Gal reporter plasmid, Petalcorin et al. (2005) showed that larvae and adult transgenic worms had nuclear *fmo* expression in intestinal cells and in hypodermal cells. Expression of *fmo-1, -2* and *-5* was mainly in intestine and also in the excretory gland region of the head, whereas *fmo-3* and* 4* were expressed in the hypodermis. In addition, hypodermal β-Gal expression was far more pronounced for *fmo-4* relative to the rest of *fmos* (Petalcorin et al. 2005), agreeing with our findings of high transcription of *C. elegans fmo-4* at especially the larval but also adult stage.

In the case of the *C. elegans fmo-1* KO strain, *fmo-2, -3* and *-5* were up-regulated genes at the larval stage. However, all remaining fmo genes were up-regulated significantly on knockout of *fmo-4*, indicating both its importance and potentially, a critical role in *C. elegans* metabolism. FMO-4 has been reported to carry out an osmoregulatory function, promoting clearance of excess water that enters during hypotonicity. This could be via the synthesis of an osmolyte that acts to establish an osmotic gradient from excretory cells to duct and pore cells (Hirani et al. 2016). *fmo-4* KO *C. elegans* had a significant decrease in egg hatching rate (data not shown), and interestingly, *fmo-4* was down-regulated in both *fmo-2* KO and *fmo-2* OE. It might have been expected to be up-regulated in the case of loss of *fmo-2*, especially as *fmo-2* was 17- to 30-fold upregulated upon loss of *fmo-4* and this warrants further investigation at the protein level in different *C. elegans* strains.

Our phenotypic analysis showed that loss of *fmo-1*, *fmo-3* and *fmo-4* statistically significantly increased *C. elegans* lifespan compared with wild type worms (Figs. 3A, B, C). These results indicate the endogenous importance of *fmo*s. It was reported that the life-extending effects of hypoxia in *C. elegans* begin in neurons with transcriptional activation by hypoxia induced factor (HIF-1) and increased serotonergic signalling (Leiser et al. 2015). These effects led to increased production of FMO-2 in the intestine, which increased longevity, whereas knocking out *fmo-2* did not affect *C. elegans* lifespan (Leiser et al. 2015). These findings agree with the results of our longevity studies here both for *fmo-2* OE (Fig. 3D) and for *fmo-2* KO (Figure S8) and of note is the fact that both studies were performed at 20 °C. Interestingly, it has also been reported that both overexpression and knockout of *fmo-2* extended lifespan at 25 °C (Leiser et al. 2011; Bennett et al. 2017). This result indicates that the *fmo-2* KO phenotype may be sensitive to temperature effects. Moreover, knockout of HIF-1 extended worm lifespan at 25 °C but not at 15 or 20 °C. Since *fmo-2* is a target of HIF-1, these results could be linked (Leiser et al. 2011, 2015), and lifespan extension upon knockout of *hif-1* at 25 °C required *daf-16* (Leiser et al. 2011; Bennett et al. 2017), thus demonstrating that longevity pathways may compensate for each other to regulate stress resistance and ageing (Leiser et al. 2011).

Loss of *fmo-4* in *C. elegans* larvae led to a dramatic ca 17- to 30-fold up-regulation of *fmo-2* transcription compared with *fmo-2* levels in wildtype (Figure S4A, 5). This could be an indication of possible compensation for the absence of *fmo-4*, and potentially, some redundancy of function. BLASTp sequence analysis of *C. elegans* FMO-2 and FMO-4 showed the lowest E-value, and the highest identity percentage (43%) and query cover (84%; Table S1), relative to any other pairwise FMO comparisons. A similar but smaller increase in *fmo-2* expression was observed in the *fmo-5* KO worm (Figure S4F,-S7).

In further agreement with our findings, in the long-lived *C. elegans* transaldolase (*tald-1)* mutant, *fmo-2* transcription was increased upon knocking down *tald-1,* by 30-to 40-fold relative to wild type. Interestingly, lifespan extension from knockdown of *tald-1* is regulated by an helix-loop-helix transcription factor (HLH-30) [orthologue of the human transcription factor EB (TFEB)] (Lin et al. 2018). TFEB is a master gene that coordinates lysosomal biogenesis by driving expression of autophagy and lysosomal genes (Settembre et al. 2011). In this *tald-1* mutant strain, *fmo-2* is upregulated in a HLH-30 and PMK-1 dependent fashion and regulates its lifespan extension (Bennett et al. 2017). *fmo-2* activity was previously shown to be necessary for lifespan extension in response to dietary restriction (DR) (Lapierre et al. 2013) and is induced by hypoxia signalling and starvation (complete bacterial food source removal) via HLH-30 (Leiser et al. 2015). However, we found no increase in *hlh-30* expression in response to *fmo-4* KO so induction and activation of FMO-2 is not clearly due to *hlh-30* upregulation (Figure S5).

A previously undescribed endogenous metabolic pathway of FMOs was recently linked with ageing processes, involving oxidation of tryptophan to form *N*-formyl-kynurenine, which is then converted to kynurenine by formamidase (Choi et al. 2021, 2023). When *fmo-2* is induced, FMO-2 oxygenates tryptophan and alters one carbon metabolism (OCM) flux by increasing formate levels, produced as a byproduct when kynurenine is synthesised from N-formyl-kynurenine (Brosnan and Brosnan 2016). It was proposed that the alteration of OCM components extended nematode lifespan by reducing methylation flux (Choi et al. 2021, 2023). The transmethylation pathway is known to affect longevity and is also involved in OCM (Choi et al. 2021, 2023). The upregulation of *fmo-2* in the absence of *fmo-4* indicates that *fmo-4* KO may extend *C. elegans* lifespan by the same mechanism as that in *fmo-2* OE, although we note that in the latter the degree of *fmo-2* up-regulation is an order of magnitude higher.

It has also been reported in *C. elegans* that intestinal lysosome-related organelles (gut granules) exhibit blue florescence which accumulate with ageing. The blue florescence was a result of altered tryptophan metabolism (from tryptophan-derived anthranilic acid glucosyl ester) through the kynurenine pathway (Coburn et al. 2013). Interestingly, in *vhl-1* mutant (long-lived) worms, the blue autofluorescence associated with age was decreased. *fmo-2* KO was reported to decrease the lifespan extension in the *vhl-1* mutant and increase the autofluorescence, whereas it has no effect on wild type worm lifespan (Leiser et al. 2015), the latter in agreement with our findings here. Moreover, FMO-2 OE decreased age-associated autofluorescence (Leiser et al. 2015). Therefore, increased tryptophan metabolite levels in *fmo-4* KO with ageing (unpublished results: manuscript in preparation) are likely associated with decreases in the blue florescence and altered metabolism of kynurenine pathway, as was found also in FMO-2 OE (Leiser et al. 2015; Choi et al. 2021).

Finally, we note that a natural product produced by the microbiome of FMO5 KO mice has recently been shown to be able to alter lipid metabolism in wildtype mice and to phenocopy elements of the FMO5 KO mice phenotype (Veeravalli et al. 2022). This finding will surely generate further interest in the critical roles that the FMOs play in endogenous mammalian metabolism.

## Conclusions

We have demonstrated here that the knockout of *fmo-1*, *fmo-3* and *fmo-4* and the over-expression of *fmo-2* significantly extends *C. elegans* lifespan relative to wild type. Our transcriptional analysis showed that in wild type worms, *fmo-1* and *fmo-4* were the most highly transcribed genes (especially at the larval stage), whereas *fmo-2* and *fmo-3* were the least transcribed, at all stages. Notably, the knockout of *fmo-4* led to a 17- to 30-fold up-regulation of *fmo-2,* along with significantly increased levels of the other *fmo*s, paralleling recent findings in the long-lived *C. elegans tald-1* mutant where *fmo-2* was also significantly up-regulated. Further experiments will be required to prove the mechanisms of lifespan extension, including the involvement of one carbon metabolism (OCM), and to explore further *fmo* roles that affect metabolic ageing. Of particular note currently, is that tryptophan and kynurenine metabolism are reported to be altered in patients suffering from acute SAR-CoV-2 infections (Thomas et al. 2020; Lawler et al. 2021) raising the possibility that the virus is also affecting FMO metabolism. Given the importance of extending healthy lifespan to the world’s ageing population, we hope that this work in some modest way will improve our understanding of the role of FMOs in the important model species, the *C. elegans* worm. Encouraging progress in this regard was recently reported by the Leiser group, in the use of FMO-induction as an early screen for pro-longevity drugs (Huang et al. 2024).

## Materials and methods

### *C. elegans* strains and maintenance

Animals were grown at 20 °C and maintained on OP50 seeded NGM plates. *C. elegans* strains were purchased from the *Caenorhabditis* Genetic Center (CGC, Minnesota, USA). *C. elegans* strains used in this study were: Bristol (N2) wild-type strain, *fmo-1* KO (RB671 [*fmo-1*(*ok405*) *IV*])*, fmo-2* KO (VC1668 [*fmo-2*(*ok2147*) *IV*]), *fmo-3* KO (RB686[*fmo-3*(ok354) III]), *fmo-4* KO (RB562 [*fmo-4*(*ok294*) *V*]), *fmo-5* KO (tm2438) and FMO-2 OE (KAE10 [*seaSi40 I; unc-119(ed3) III*]). These mutants (*fmo-1-fmo-5* KO) were verified by the absence of transcription of their corresponding genes using RT-PCR (see Supplementary Figures S9-S14).

### Lifespan

Survival assays were carried out in 60 mm (Fisher) Petri dishes. 120 µl of OP50 were transferred to the centre of each 60 mm NGM plate and 220 µl of OP50 was added to each 90 mm NGM plate, then incubated at 20 °C for 2 days (Sutphin and Kaeberlein 2009; Amrit et al. 2014). 100 µl of 500 µM floxuridine (FUdR; Carbosynth, Staad, Switzerland) was then added to the centre of each NGM plate.

Synchronised cultures of each strain were used. Eggs of each strain were transferred to 90 mm OP50 NGM seeded plates. After 2 days of incubation worms were checked under the microscope for growth, when they reached larvae 4 (L4) stage, they were transferred to labelled FUdR loaded NGM 60 mm plates (Four plates per group, 100 worms per plate). Worms were checked and counted directly after the transfer (Stiernagle 2006; Sutphin and Kaeberlein 2009).

NGM plates of each strain were examined under a dissecting microscope every 2–3 days, recording live and dead worms. Worms were transferred to new seeded plates every 2–3 days until they reached day 12 post-hatching. Moving worms were recorded as live, but immobile worms were checked by tapping on the worm head to initiate motility. Worms which responded to tapping were recorded as alive, whereas worms which did not respond were recorded as dead. Worms that crawled off the plates or were damaged during worm transfer were recorded as censored. Survival data were analysed using Kaplan–Meier estimate (Sutphin and Kaeberlein 2009). Survival curves were plotted with the GraphPad Prism 6 software and statistical analyses were performed using the log-rank method.

### qRT-PCR

50 mg of synchronised cultures of embryo, larvae (L1) and day 1 adult worms were collected and frozen in Trizol reagents (ThermoFisher, Massachusetts, USA). Worm pellets were transferred to 2 ml prefilled micro centrifuge screw tube containing 0.1 mm (0.25 g) and 1 mm zirconium beads (0.25 g; Sigma, Poole, UK) with 1 ml Trizol (Fisher) and the tube was shaken using a TissueLyser II (Qiagen, Hilden, Germany) for 7 min at 30 Hz. 400 μl of chloroform was added, shaken vigorously (15 s) and incubated at RT for 3 min. Samples were then spun at 12.000 *g* for 15 min at 4 °C. Aqueous phase was collected and RNA was purified using RNeasy mini kit (Qiagen). Genomic DNA was removed using On-Column DNase (Sigma). Purity of eluted RNA was estimated by OD ratios (A260/A280 > 2.0) and quantified using the BioDrop spectrophotometer (Scientific lab supplies, Nottingham, UK; Table S2), then stored at − 80 °C until needed. RNA integrity was tested by running all RNA samples on gel electrophoresis, intact RNA samples showing two clear bands for 18S and 28S rRNA (Figure S15–S16). 1 µg total RNA from each sample was converted into cDNA using RevertAid First Strand cDNA Synthesis Kit (ThermoFisher) with oligo (dT) primers as reverse transcription primers. Three biological samples were analysed in triplicate using gene specific primers (Table S3-S4). Transcription levels were determined using QuantStudio1 system (Applied Biosystems, California, USA) with the SYBR Green probe (Qiagen). Prepared cDNA was amplified under the following conditions: 1 µl of cDNA, 5 µl of SYBR Green PCR master mix (ThermoFisher), 1 µl of each forward/reverse primer (10 pmol) and 3 µl of H_2_O per 10 µl reaction. The PCR conditions were as follows: initial denaturation at 95 °C for 5 min, 40 cycles of 95 °C for 1 min and 60 °C for 30 s. Primers were designed using OligoArchitect (Premier Biosoft International, California, USA) to produce amplicons between 100 and 200 bp. PCR and gel electrophoresis with the prepared cDNA was used to verify primer specificity and to rule out any genomic DNA contamination (Figure S17). Melt curves were analysed to ensure single melt curve peaks were produced (Figure S18). Primer efficiency (E) and primer stability (M) were determined over tenfold serial dilutions of cDNA (Figure S19-S21). Primer efficiencies were determined using E = (10(− 1/slope) − 1) * 100, high amplification efficiency being between 90 and 110% (Table S5). In this study, five HK genes (*pmp-3*, F35G12.2, *tba-1*, Y45F10D.4 and *rbd-1*) were evaluated for their stability using the four commonly used algorithms geNorm (Hoogewijs et al. 2008), NormFinder (Andersen et al. 2004), the comparative delta *Ct* method (Silver et al. 2006),and BestKeeper (Pfaffl et al. 2004) to validate HK gene selection. Comprehensive ranking of the HK genes using four different algorithms indicated that *pmp-3* and F35G12.2 were the most two stable HK genes. Also, their transcription throughout development stages was stable (Figure S22, S23). *Pmp-3* and F35G12.2 were thus used for normalisation that was done by calculating the mean of *Ct* values of the two HK genes at each stage. The mean was used in the comparative delta *Ct* equations instead of the mean of one HK gene (Livak and Schmittgen 2001). Data were analysed using Graphpad Prism 6.0 software (La Jolla, California, USA). Each experiment consisted of three biological replicates. Graphs were plotted to show mean (± SEM) of population, and subjected to either student *t* tests or one-way ANOVA and post-hoc Dunnett tests (Kim 2015) to determine statistically significant differences of gene transcription between WT and *fmo* knockouts.

### RT-PCR

1 µg total RNA from each sample was converted into cDNA using RevertAid First Strand cDNA Synthesis Kit (ThermoFisher) with oligo (dT) primers as reverse transcription primers. Prepared cDNA was amplified under the following conditions: 1 µl of cDNA, 12.5 µl of DreamTaq buffer (ThermoFisher), 2 µl of each forward/reverse primer (10 pmol) and 9.5 µl of H_2_O per 25 µl reaction. Amplification of genes was performed using thermocycler (Bio-Rad, California, USA). The PCR conditions were as follows: initial denaturation, 98 °C for 1 min, 30 cycles of 98 °C for 5 s, 54 °C for 15 s and 72 °C for 30 s, then final extension at 72 °C for 5 min. Primers were designed using primer blast (https://www.ncbi.nlm.nih.gov/tools/primer-blast/) and supplied by Sigma (Table S6). Gel electrophoresis of the amplicons was used to verify the mutants (Figure S9–S14).

## Supplementary Information

Below is the link to the electronic supplementary material.

Supplementary file1 (PDF 4322 KB)

## Data Availability

The datasets generated during and/or analysed during the current study are available from the corresponding authors on reasonable request.

## References

[CR1] Altun ZF, Hall DH (2009) Introduction. WormAtlas. 10.3908/wormatlas.1.1

[CR2] Amrit FRG, Ratnappan R et al (2014) The *C. elegans* lifespan assay toolkit. Methods 68(3):465–475. 10.1016/j.ymeth.2014.04.002 24727064 10.1016/j.ymeth.2014.04.002

[CR3] Andersen CL, Jensen JL et al (2004) Normalization of real-time quantitative reverse transcription-PCR data: a model-based variance estimation approach to identify genes suited for normalization, applied to bladder and colon cancer data sets. Can Res 64(15):5245–5250. 10.1158/0008-5472.can-04-0496 10.1158/0008-5472.CAN-04-0496 15289330

[CR4] Bennett CF, Kwon JJ et al (2017) Transaldolase inhibition impairs mitochondrial respiration and induces a starvation-like longevity response in *Caenorhabditis elegans*. PLoS Genet 13(3):e1006695. 10.1371/journal.pgen.1006695 28355222 10.1371/journal.pgen.1006695 PMC5389855

[CR5] Brosnan ME, Brosnan JT (2016) Formate: the neglected member of one-carbon metabolism. Annu Rev Nutr 36:369–388. 10.1146/annurev-nutr-071715-050738 27431368 10.1146/annurev-nutr-071715-050738

[CR6] Chen Y, Patel NA et al (2011) Bacterial flavin-containing monooxygenase is trimethylamine monooxygenase. Proc Natl Acad Sci USA 108(43):17791–17796. 10.1073/pnas.1112928108 22006322 10.1073/pnas.1112928108 PMC3203794

[CR7] Choi HS, Bhat A et al (2021) FMO rewires metabolism to promote longevity through tryptophan and one carbon metabolism. bioRxiv: 2021.2006.2018.449022. 10.1101/2021.06.18.449022

[CR8] Choi HS, Bhat A et al (2023) FMO rewires metabolism to promote longevity through tryptophan and one carbon metabolism in *C. elegans*. Nat Commun 14:562. 10.1038/s41467-023-36181-0 36732543 10.1038/s41467-023-36181-0 PMC9894935

[CR9] Coburn C, Allman E et al (2013) Anthranilate fluorescence marks a calcium-propagated necrotic wave that promotes organismal death in *C. elegans*. PLoS Biol 11(7):e1001613. 10.1371/journal.pbio.1001613 23935448 10.1371/journal.pbio.1001613 PMC3720247

[CR10] Corsi AK, Wightman B et al (2015) A transparent window into biology: a primer on *Caenorhabditis elegans*. Genetics 200(2):387–407. 10.1534/genetics.115.176099 26088431 10.1534/genetics.115.176099 PMC4492366

[CR11] Dolphin CT, Janmohamed A et al (1997) Missense mutation in flavin-containing mono-oxygenase 3 gene, FMO3, underlies fish-odour syndrome. Nat Genet 17(4):491–494. 10.1038/ng1297-491 9398858 10.1038/ng1297-491

[CR12] Gujar MR, Stricker AM et al (2017) Flavin monooxygenases regulate *Caenorhabditis elegans* axon guidance and growth cone protrusion with UNC-6/Netrin signaling and Rac GTPases. PLoS Genet 13(8):26. 10.1371/journal.pgen.1006998 10.1371/journal.pgen.1006998 PMC5597259 28859089

[CR13] Hernandez D, Janmohamed A et al (2004) Organization and evolution of the flavin-containing monooxygenase genes of human and mouse: identification of novel gene and pseudogene clusters. Pharmacogenetics 14(2):117–130. 10.1097/00008571-200402000-00006 15077013 10.1097/00008571-200402000-00006

[CR14] Hirani N, Westenberg M et al (2016) *C. elegans* flavin-containing monooxygenase-4 is essential for osmoregulation in hypotonic stress. Biology Open 5(5):537–549. 10.1242/bio.017400 27010030 10.1242/bio.017400 PMC4874355

[CR15] Hoogewijs D, Houthoofd K et al (2008) Selection and validation of a set of reliable reference genes for quantitative sod gene expression analysis in *C. elegans*. BMC Mol Biol. 10.1186/1471-2199-9-9 18211699 10.1186/1471-2199-9-9 PMC2254638

[CR16] Huang S, Howington MB et al (2021) Flavin-containing monooxygenases are conserved regulators of stress resistance and metabolism. Front Cell Dev Biol 9:151 10.3389/fcell.2021.630188 PMC7907451 33644069

[CR17] Huang S, Cox RL et al (2024) Fmo induction as a tool to screen for pro-longevity drugs. GeroScience. 10.1007/s11357-024-01207-y 38787463 10.1007/s11357-024-01207-y PMC11335711

[CR18] Ivan M, Kaelin WG Jr (2001) The von Hippel–Lindau tumor suppressor protein. Curr Opin Genet Dev 11(1):27–34. 10.1016/s0959-437x(00)00152-0 11163147 10.1016/s0959-437x(00)00152-0

[CR19] Jiang H, Guo R et al (2001) The *Caenorhabditis elegans* hif-1 gene encodes a bHLH-PAS protein that is required for adaptation to hypoxia. Proc Natl Acad Sci U S A 98(14):7916–7921. 10.1073/pnas.141234698 11427734 10.1073/pnas.141234698 PMC35443

[CR20] Kim HY (2015) Statistical notes for clinical researchers: post-hoc multiple comparisons. Restor Dent Endod 40(2):172–176. 10.5395/rde.2015.40.2.172 25984481 10.5395/rde.2015.40.2.172 PMC4432262

[CR21] Krueger SK, Williams DE (2005) Mammalian flavin-containing monooxygenases: structure/function, genetic polymorphisms and role in drug metabolism. Pharmacol Ther 106(3):357–387. 10.1016/j.pharmthera.2005.01.001 15922018 10.1016/j.pharmthera.2005.01.001 PMC1828602

[CR22] Lapierre LR, De Magalhaes Filho CD et al (2013) The TFEB orthologue HLH-30 regulates autophagy and modulates longevity in *Caenorhabditis elegans*. Nat Commun 4:2267. 10.1038/ncomms3267 23925298 10.1038/ncomms3267 PMC3866206

[CR23] Lawler NG, Gray N et al (2021) Systemic perturbations in amine and kynurenine metabolism associated with acute SARS-CoV-2 infection and inflammatory cytokine responses. J Proteome Res 20(5):2796–2811. 10.1021/acs.jproteome.1c00052 33724837 10.1021/acs.jproteome.1c00052

[CR24] Leiser SF, Begun A et al (2011) HIF-1 modulates longevity and healthspan in a temperature-dependent manner. Aging Cell 10(2):318–326. 10.1111/j.1474-9726.2011.00672.x 21241450 10.1111/j.1474-9726.2011.00672.x PMC3980873

[CR25] Leiser SF, Miller H et al (2015) Cell nonautonomous activation of flavin-containing monooxygenase promotes longevity and health span. Science 350(6266):1375–1378. 10.1126/science.aac9257 26586189 10.1126/science.aac9257 PMC4801033

[CR26] Lin X-X, Sen I et al (2018) DAF-16/FOXO and HLH-30/TFEB function as combinatorial transcription factors to promote stress resistance and longevity. Nat Commun 9(1):4400. 10.1038/s41467-018-06624-0 30353013 10.1038/s41467-018-06624-0 PMC6199276

[CR27] Livak KJ, Schmittgen TD (2001) Analysis of relative gene expression data using real-time quantitative PCR and the 2(-Delta Delta C(T)) Method. Methods 25(4):402–408. 10.1006/meth.2001.1262 11846609 10.1006/meth.2001.1262

[CR28] Malagon SGG, Melidoni AN et al (2015) The phenotype of a knockout mouse identifies flavin-containing monooxygenase 5 (FMO5) as a regulator of metabolic ageing. Biochem Pharmacol 96(3):267–277. 10.1016/j.bcp.2015.05.013 26049045 10.1016/j.bcp.2015.05.013 PMC4509511

[CR29] Nicoll CR, Bailleul G et al (2020) Ancestral-sequence reconstruction unveils the structural basis of function in mammalian FMOs. Nat Struct Mol Biol 27(1):14–24. 10.1038/s41594-019-0347-2 31873300 10.1038/s41594-019-0347-2

[CR30] Petalcorin MIR, Joshua GW et al (2005) The FMO genes of *Caenorhabditis elegans* and *C. briggsae*: characterisation, gene expression and comparative genomic analysis. Gene 346:83–96. 10.1016/j.gene.2004.09.021 15716098 10.1016/j.gene.2004.09.021

[CR31] Pfaffl MW, Tichopad A et al (2004) Determination of stable housekeeping genes, differentially regulated target genes and sample integrity: BestKeeper - excel-based tool using pair-wise correlations. Biotech Lett 26(6):509–515. 10.1023/b:bile.0000019559.84305.47 10.1023/b:bile.0000019559.84305.47 15127793

[CR32] Phillips IR, Shephard EA (2008) Flavin-containing monooxygenases: mutations, disease and drug response. Trends Pharmacol Sci 29(6):294–301. 10.1016/j.tips.2008.03.004 18423897 10.1016/j.tips.2008.03.004

[CR33] Phillips IR, Shephard EA (2017) Drug metabolism by flavin-containing monooxygenases of human and mouse. Expert Opin Drug Metab Toxicol 13(2):167–181. 10.1080/17425255.2017.1239718 27678284 10.1080/17425255.2017.1239718

[CR34] Phillips IR, Shephard EA (2020) Flavin-containing monooxygenase 3 (FMO3): genetic variants and their consequences for drug metabolism and disease. Xenobiotica 50(1):19–33. 10.1080/00498254.2019.1643515 31317802 10.1080/00498254.2019.1643515

[CR35] Robert X, Gouet P (2014) Deciphering key features in protein structures with the new ENDscript server. Nucleic Acids Res 42(Web Server issue):W320-324. 10.1093/nar/gku316 24753421 10.1093/nar/gku316 PMC4086106

[CR36] Rossner R, Kaeberlein M et al (2017) Flavin-containing monooxygenases in aging and disease: emerging roles for ancient enzymes. J Biol Chem 292(27):11138–11146. 10.1074/jbc.R117.779678 28515321 10.1074/jbc.R117.779678 PMC5500783

[CR37] Settembre C, Di Malta C et al (2011) TFEB links autophagy to lysosomal biogenesis. Science 332(6036):1429–1433. 10.1126/science.1204592 21617040 10.1126/science.1204592 PMC3638014

[CR38] Shephard EA, Treacy EP et al (2012) Clinical utility gene card for: trimethylaminuria. Eur J Hum Genet 20(3):4–5. 10.1038/ejhg.2011.214 22126753 10.1038/ejhg.2011.214 PMC3283181

[CR39] Sievers F, Wilm A et al (2011) Fast, scalable generation of high-quality protein multiple sequence alignments using Clustal Omega. Mol Syst Biol 7:539. 10.1038/msb.2011.75 21988835 10.1038/msb.2011.75 PMC3261699

[CR40] Silver N, Best S et al (2006) Selection of housekeeping genes for gene expression studies in human reticulocytes using real-time PCR. BMC Mol Biol. 10.1186/1471-2199-7-33 17026756 10.1186/1471-2199-7-33 PMC1609175

[CR41] Stiernagle T (2006) Maintenance of *C. elegans*, W*ormBook*, ed. The C*. elegans* Research Community, WormBook. 10.1895/wormbook.

[CR42] Sutphin GL, Kaeberlein M (2009) Measuring *Caenorhabditis elegans* life span on solid media. JoVE. 10.3791/1152 PMC2794294 19488025

[CR43] Thomas T, Stefanoni D et al (2020) COVID-19 infection alters kynurenine and fatty acid metabolism, correlating with IL-6 levels and renal status. JCI Insight. 10.1172/jci.insight.140327 32559180 10.1172/jci.insight.140327 PMC7453907

[CR44] Uno M, Nishida E (2016) Lifespan-regulating genes in *C. elegans*. Npj Aging Mech Dis 2:8. 10.1038/npjamd.2016.10 10.1038/npjamd.2016.10 PMC5514992 28721266

[CR45] Veeravalli S, Phillips IR et al (2020) Flavin-containing monooxygenase 1 (FMO1) catalyzes the production of taurine from hypotaurine. Drug Metab Dispos Biol Fate Chem. 10.1124/dmd.119.089995 32156684 10.1124/dmd.119.089995

[CR46] Veeravalli S, Varshavi D et al (2022) Treatment of wild-type mice with 2,3-butanediol, a urinary biomarker of Fmo5(-/-) mice, decreases plasma cholesterol and epididymal fat deposition. Front Physiol. 10.3389/fphys.2022.859681 36003643 10.3389/fphys.2022.859681 PMC9393927

[CR47] Yamazaki H, Shimizu M (2007) Genetic polymorphism of the flavin-containing monooxygenase 3 (FMO3) associated with trimethylaminuria (fish odor syndrome): observations from Japanese patients. Curr Drug Metab 8(5):487–491. 10.2174/138920007780866825 17584019 10.2174/138920007780866825

[CR48] Ziegler DM (2002) An overview of the mechanism, substrate specificities, and structure of FMOs. Drug Metab Rev 34(3):503–511. 10.1081/dmr-120005650 12214662 10.1081/dmr-120005650

