# Supplementary material for: Transcriptional analysis of C. elegans fmos at different life stages and their roles in ageing
Source: Mol Genet Genomics. 2024 Dec 5;299(1):113. doi: 10.1007/s00438-024-02201-x (PMC11621177; doi:10.1007/s00438-024-02201-x)
Supplement: Supplementary file 1 — Supplementary file1 (PDF 4322 KB) [file 438_2024_2201_MOESM1_ESM.pdf]

Transcriptional analysis of *C. elegans fmo*s at different life stages and their roles in ageing

Supplementary Materials

Authors

Mohamed Said[1,#], Bill T. Ferrara[1], Andreea Aprodu[3], Filipe Cabreiro[2,3], Elinor P. Thompson*[1], Jeremy Everett*[1]

1- Faculty of Engineering and Science, University of Greenwich, Chatham Maritime, Kent ME4 4TB, UK

2- Institute of Clinical Sciences, Imperial College London W12 0NN, UK

3 - Cologne Excellence Cluster for Cellular Stress Responses in Aging-Associated Diseases (CECAD), University of Cologne, Joseph Stelzmann Strasse 26, 50931 Cologne, Germany

# - Current Address, Faculty of Pharmacy, October University for Modern Sciences and Arts, 6th October City, Egypt

* Corresponding authors: j.r.everett@greenwich.ac.uk, ORCID ID: 0000-0003-1550-4482 and e.thompson@greenwich.ac.uk, ORCID ID: 0000-0002-6434-9290

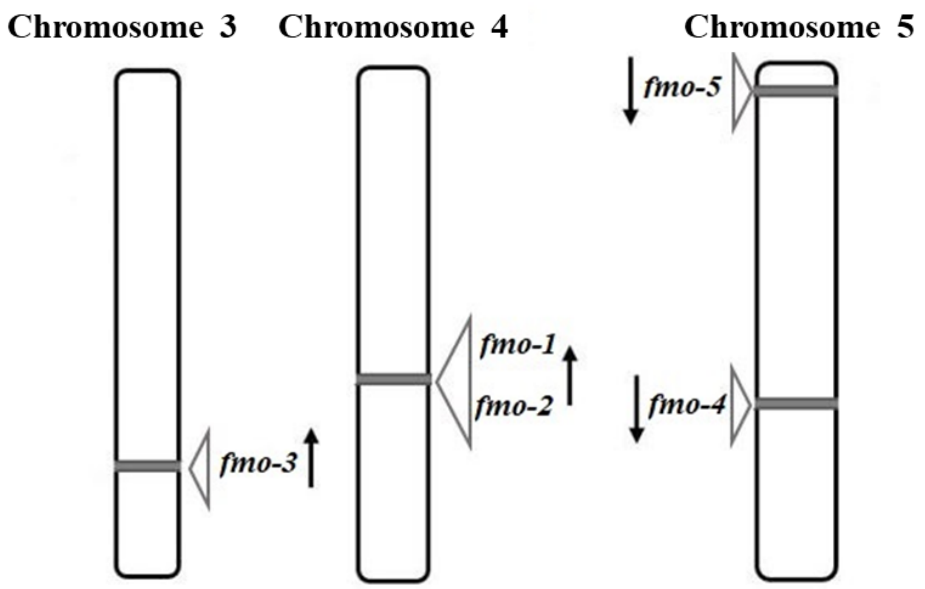

Figure S1. *fmo* genes location on *C. elegans* chromosomes.

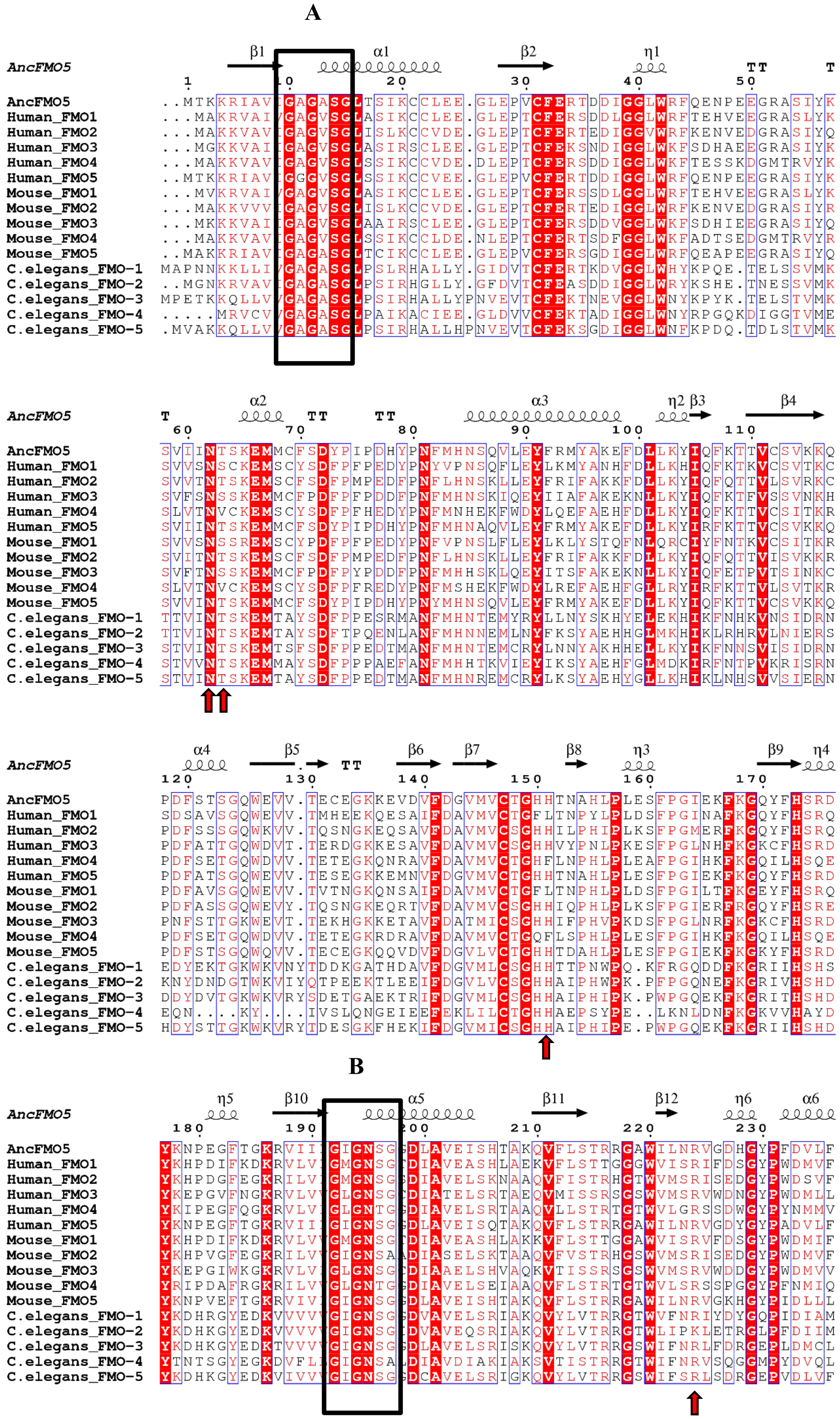
A
B
AncFMO5
β1 α1 β2 η1 TT T
1 10 20 30 40 50
AncFMO5 ..MTKKRIAVIGAGASGLTSIKCCLEE.GLEPVCFERTDDIGGLWRFQENPEEGRASIYK
Human_FMO1 ...MAKRVAIVGAGVSGLASIKCCLEE.GLEPTCFERSDDLGGLWRFTEHVEEGRASLYK
Human_FMO2 ...MAKKVAVIGAGVSGLISLKCCVDE.GLEPTCFERTEDIGGVWRFKENVEDGRASIYQ
Human_FMO3 ...MGKKVAIIGAGVSGLASIRSCLEE.GLEPTCFEKSNDIGGLWKFSDHAEEGRASIYK
Human_FMO4 ...MAKKVAVIGAGVSGLSSIKCCVDE.DLEPTCFERSDDIGGLWKFTESSKDGMTRVYK
Human_FMO5 ..MTKKRIAVIGGGVSGLSSIKCCVEE.GLEPVCFERTDDIGGLWRFQENPEEGRASIYK
Mouse_FMO1 ...MVKRVAIVGAGVSGLASIKCCLEE.GLEPTCFERSSDLGGLWRFTEHVEEGRASLYK
Mouse_FMO2 ...MAKKVVVIGAGVSGLISLKCCVDE.GLEPTCFERTEDIGGLWRFKENVEDGRASIYR
Mouse_FMO3 ...MKKKVAIIGAGVSGLAAIRSCLEE.GLEPTCFERSDDVGGLWKFSDHIEEGRASIYQ
Mouse_FMO4 ...MAKKVAVIGAGVSGLSSIKCCLDE.NLEPTCFERTSDFGGLWKFADTSEDGMTRVYR
Mouse_FMO5 ..MAKKRIAVIGAGASGLTCIKCCLEE.GLEPVCFERSGDIGGLWRFQEAPEEGRASIYQ
C.elegans_FMO-1 MAPNNKKLLIIGAGASGLPSLRHALLY.GIDVTCFERTKDVGGLWHYKPQE.TELSSVMK
C.elegans_FMO-2 ..MGNKRVAVIGAGASGLPSIRHGLLY.GFDVTCFEASDDIGGLWRYKSHE.TNESSVMK
C.elegans_FMO-3 MPETKKQLLVVGAGASGLPSIRHALLYPNVEVTCFEKTNEVGGLWNYKPYK.TELSTVMK
C.elegans_FMO-4 .....MRVCVVGAGASGLPAIKACIEE.GLDVVCFEKTADIGGLWNYRPGQKDIGGTVME
C.elegans_FMO-5 .MVAKKQLLVVGAGASGLPSIRHALLHPNVEVTCFEKSGDIGGLWNFKPDQ.TDLSTVMK
AncFMO5
T α2 TT TT α3 η2 β3 β4
60 70 80 90 100 110
AncFMO5 SVIINTSKEMMCFSDYPIPDHYPNFMHNSQVLEYFRMYAKEFDLLKYIQFKTTVCSVKKQ
Human_FMO1 SVVSNSCKEMSCYSDFPFPEDYPNYVPNSQFLEYLKMYANHFDLLKHIQFKTKVCSVTKC
Human_FMO2 SVVTNTSKEMSCFSDFPMPEDFPNFLHNSKLLEYFRIFAKKFDLLKYIQFQTTVLSVRKC
Human_FMO3 SVFSNSSKEMMCFPDFPFPDDFPNFMHNSKIQEYIIAFAKEKNLLKYIQFKTFVSSVNKH
Human_FMO4 SLVTNVCKEMSCYSDFPFHEDYPNFMNHEKFWDYLQEFAEHFDLLKYIQFKTTVCSITKR
Human_FMO5 SVIINTSKEMMCFSDYPIPDHYPNFMHNAQVLEYFRMYAKEFDLLKYIRFKTTVCSVKKQ
Mouse_FMO1 SVVSNSSREMSCYPDFPFPEDYPNFVPNSLFLEYLKLYSTQFNLQRCIYFNTKVCSITKR
Mouse_FMO2 SVITNTSKEMSCFSDFPMPEDFPNFLHNSKLLEYFRIFAKKFDLLKYIQFQTTVISVKKR
Mouse_FMO3 SVFTNSSKEMMCFPDFPYPDDFPNFMHHSKLQEYITSFAKEKNLLKYIQFETPVTSINKC
Mouse_FMO4 SLVTNVCKEMSCYSDFPFREDYPNFMSHEKFWDYLREFAEHFGLLRYIRFKTTVLSVTKR
Mouse_FMO5 SVVINTSKEMMCFSDYPIPDHYPNYMHNSQVLEYFRMYAKEFDLLKYIQFKTTVCSVKKQ
C.elegans_FMO-1 TTVINTSKEMTAYSDFPPESRMANFMHNTEMYRYLLNYSKHYELEKHIKFNHKVNSIDRN
C.elegans_FMO-2 TTVINTSKEMTAYSDFTPQENLANFMHNNEMLNYFKSYAEHHGLMKHIKLRHRVLNIERS
C.elegans_FMO-3 STVINTSKEMTSFSDFPPEDTMANFMHNTEMCRYLNNYAKHHELLKYIKFNNSVISIDRN
C.elegans_FMO-4 STVVNTSKEMMAYSDFPPPAEFANFMHHTKVIEYIKSYAEHFGLMDKIRFNTPVKRISRN
C.elegans_FMO-5 STVINTSKEMTAYSDFPPEDTMANFMHNREMCRYLKSYAEHYGLLKHIKLNHSVVSIERN
AncFMO5
α4 β5 TT β6 β7 β8 η3 β9 η4
120 130 140 150 160 170
AncFMO5 PDFSTSGQWEVV.TECEGKKEVDVFDGVMVCTGHHTNAHLPLESFPGIEKFKGQYFHSRD
Human_FMO1 SDSAVSGQWEVV.TMHEEKQESAIFDAVMVCTGFLTNPYLPLDSFPGINAFKGQYFHSRQ
Human_FMO2 PDFSSSGQWKVV.TQSNGKEQSAVFDAVMVCSGHHILPHIPLKSFPGMERFKGQYFHSRQ
Human_FMO3 PDFATTGQWDVT.TERDGKKESAVFDAVMVCSGHHVYPNLPKESFPGLNHFKGKCFHSRD
Human_FMO4 PDFSETGQWDVV.TETEGKQNRAVFDAVMVCTGHFLNPHLPLEAFPGIHKFKGQILHSQE
Human_FMO5 PDFATSGQWEVV.TESEGKKEMNVFDGVMVCTGHHTNAHLPLESFPGIEKFKGQYFHSRD
Mouse_FMO1 PDFAVSGQWEVV.TVTNGKQNSAIFDAVMVCTGFLTNPHLPLDSFPGILTFKGEYFHSRQ
Mouse_FMO2 PDFASSGQWEVY.TQSNGKEQRTVFDAVMVCSGHHIQPHLPLKSFPGIERFRGQYFHSRE
Mouse_FMO3 PNFSTTGKWEVT.TEKHGKKETAVFDATMICSGHHIFPHVPKDSFPGLNRFKGKCFHSRD
Mouse_FMO4 PDFSETGQWDVV.TETEGKRDRAVFDAVMVCTGQFLSPHLPLESFPGIHKFKGQILHSQE
Mouse_FMO5 PDFSTSGQWQVV.TECEGKQQVDVFDGVLVCTGHHTDAHLPLESFPGIEKFKGKYFHSRD
C.elegans_FMO-1 EDYEKTGKWKVNYTDDKGATHDAVFDGVMLCSGHHTTPNWPQ.KFRGQDDFKGRIIHSHS
C.elegans_FMO-2 KNYDNDGTWKVIYQTPEEKTLEEIFDGVLVCSGHHAIPHWPK.PFPGQNEFKGRIVHSHD
C.elegans_FMO-3 DDYDVTGKWKVRYSDETGAEKTRIFDGVMLCSGHHAIPHIPK.PWPGQEKFKGRITHSHD
C.elegans_FMO-4 EQN....KY....IVSLQNGEIEEFEKLILCTGHHAEPSYPE..LKNLDNFKGKVVHAYD
C.elegans_FMO-5 HDYSTTGKWKVRYTDESGKFHEKIFDGVMICSGHHAIPHIPE.PWPGQEKFKGRIIHSHD
AncFMO5
η5 β10 α5 β11 β12 η6 α6
180 190 200 210 220 230
AncFMO5 YKNPEGFTGKRVIIIGIGNSGGDLAVEISHTAKQVFLSTRRGAWILNRVGDHGYPFDVLF
Human_FMO1 YKHPDIFKDKRVLVIGMGNSGTDIAVEASHLAEKVFLSTTGGGWVISRIFDSGYPWDMVF
Human_FMO2 YKHPDGFEGKRILVIGMGNSGSDIAVELSKNAAQVFISTRHGTWVMSRISEDGYPWDSVF
Human_FMO3 YKEPGVFNGKRVLVVGLGNSGCDIATELSRTAEQVMISSRSGSWVMSRVWDNGYPWDMLL
Human_FMO4 YKIPEGFQGKRVLVIGLGNTGGDIAVELSRTAAQVLLSTRTGTWVLGRSSDWGYPYNMMV
Human_FMO5 YKNPEGFTGKRVIIIGIGNSGGDLAVEISQTAKQVFLSTRRGAWILNRVGDYGYPADVLF
Mouse_FMO1 YKHPDIFKDKRVLVVGMGNSGTDIAVEASHLAKKVFLSTTGGAWVISRVFDSGYPWDMIF
Mouse_FMO2 YKHPVGFEGKRILVIGIGNSAADIASELSKTAAQVFVSTRHGSWVMSRISEDGYPWDMVF
Mouse_FMO3 YKEPGIWKGKRVLVIGLGNSGCDIAAELSHVAQKVTISSRSGSWVMSRVWDDGYPWDMVV
Mouse_FMO4 YRIPDAFRGKRILVIGLGNTGGDIAVELSEIAAQVFLSTRTGTWVLSRSSPGGYPFNMIQ
Mouse_FMO5 YKNPVEFTGKRVIVIGLGNSGGDLAVEISHTAKQVFLSTRRGAWILNRVGKHGYPIDLLL
C.elegans_FMO-1 YKDHRGYEDKVVVVIGIGNSGGDVAVELSRIAKQVYLVTRRGTWVFNRIYDYGQPIDIAM
C.elegans_FMO-2 YKDHKGYEDKVVVVIGIGNSGIDVAVEQSRIAKQVYLVTRRGTWLIPKLETRGLPFDIIM
C.elegans_FMO-3 YKDHKGYEDKTVVVIGIGNSGGDLAVELSRISKQVYLVTRRGSWIFNRLFDRGEPLDMCL
C.elegans_FMO-4 YTNTSGYEGKDVFLIGIGNSAADIAVDIAKIAKSVTISTRRGTWIFNRVSQGGMPYDVQL
C.elegans_FMO-5 YKDHKGYEDKVIVVIGIGNSGGDCAVELSRIGKQVYLVTRRGSWIFSRLSDRGEPVDLVF

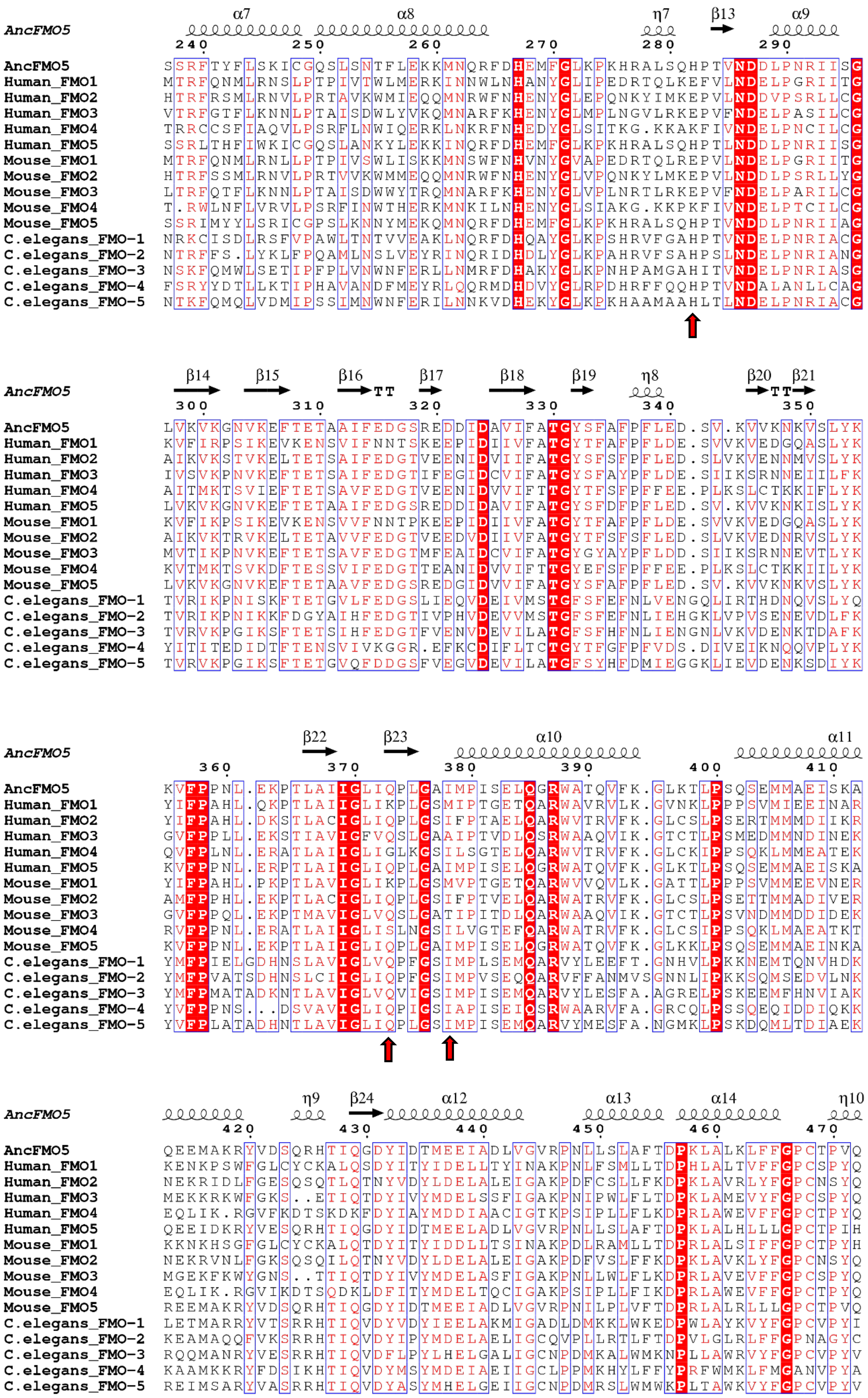

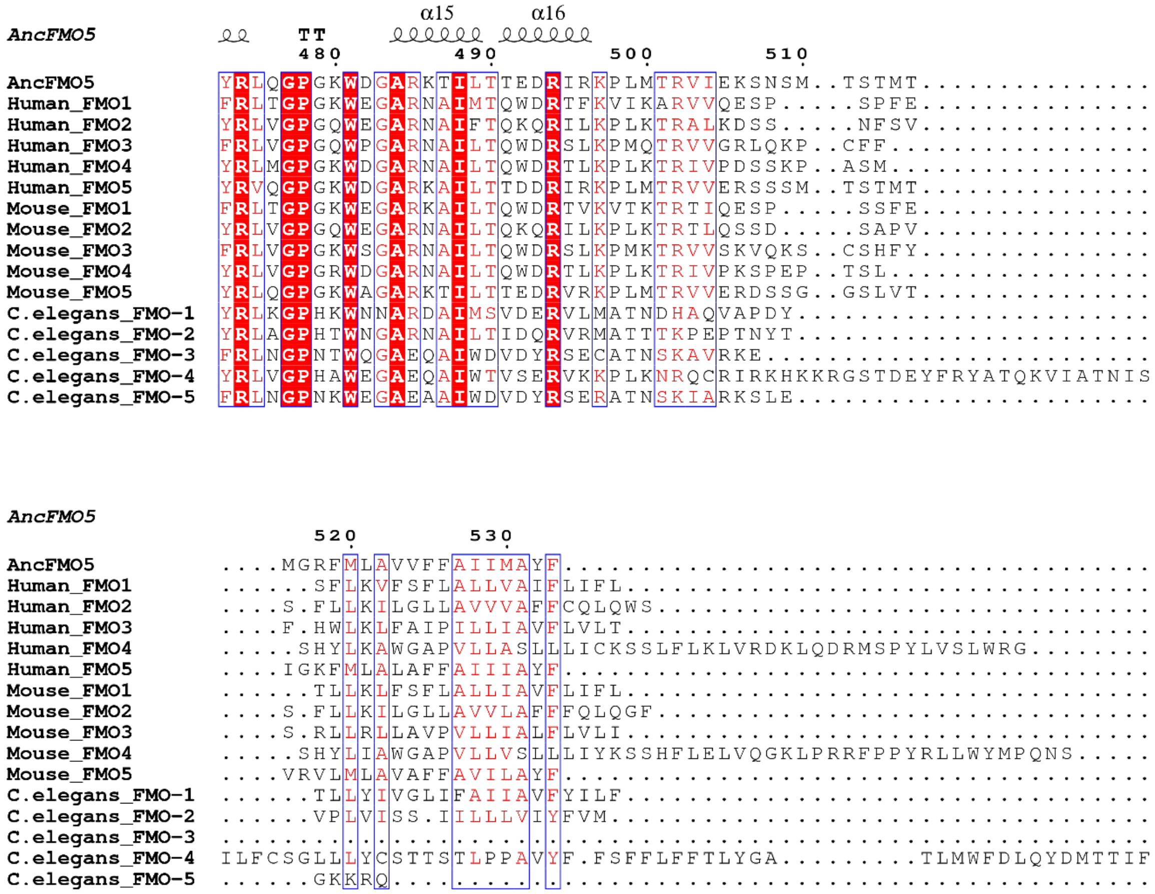

Figure S2. Amino acid sequence alignment of FMOs across species with the regions containing essential FAD and NADP binding domains and the eight essential residues denoted as red arrowheads in the catalytic active site among reconstructed ancestral mammalian FMOs. **A**, Sequences important for FAD binding; **B**, Sequences important for NADP binding. Alignment figures were generated using Clustal Omega (Sievers et al., 2011) and and ESPript 3.0 (Robert and Gouet, 2014)

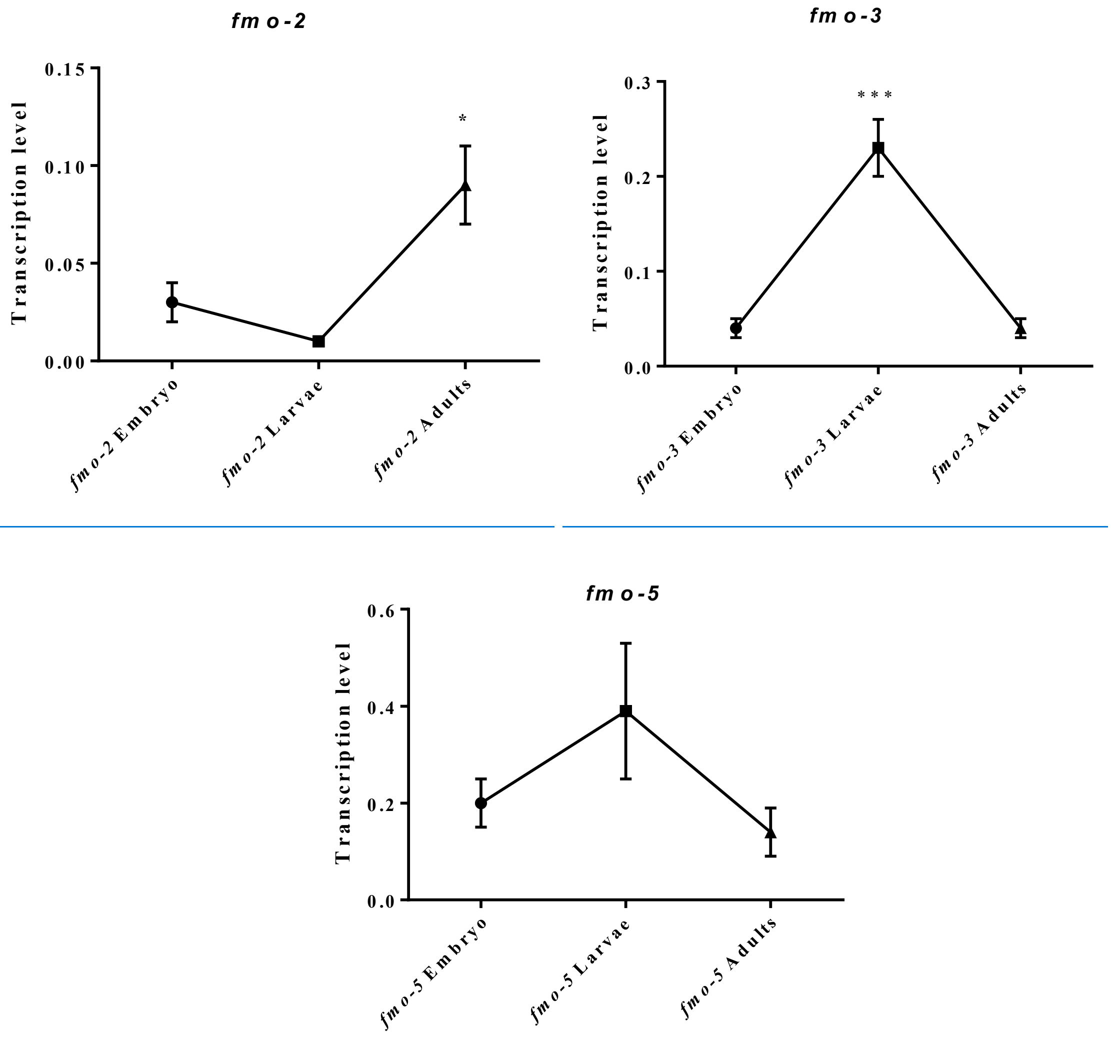

Figure S3: Transcriptional analysis of the *fmo-2,-3* and *-5* in embryo, larvae (L2; day 1 post hatching) and adult wild type *C. elegans* using qRT-PCR. 2B. Mean (± SEM) analysed from three biological repeats, normalised to the HK genes pmp-3 and F35G12.2. HK, Housekeeping genes. Each analysis was run in triplicate. One-way ANOVA using post hoc Tukey test: ns, $P > 0.05$; *, $P < 0.05$; **, $P < 0.01$; ***, $P < 0.001$.

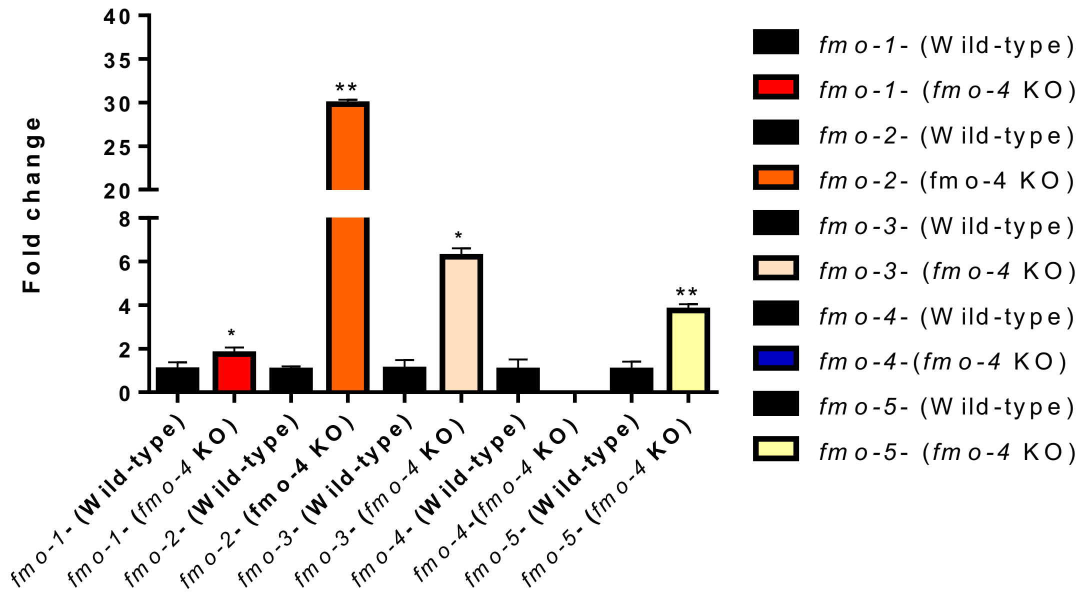

Figure S4A: Transcriptional analysis of the *fmo* genes (*fmo1-5*) in larvae (L2; day 1 post hatching), wild type and *fmo-4* KO *C. elegans* using RT-qPCR. Mean (± SEM) analysed from 3 biological repeats, normalised to the HK gene pmp3 and F35G12.2. HK, Housekeeping genes; KO, knockout; each reaction was run in triplicate. One-way ANOVA: ns, P > 0.05; ***, P < 0.001.

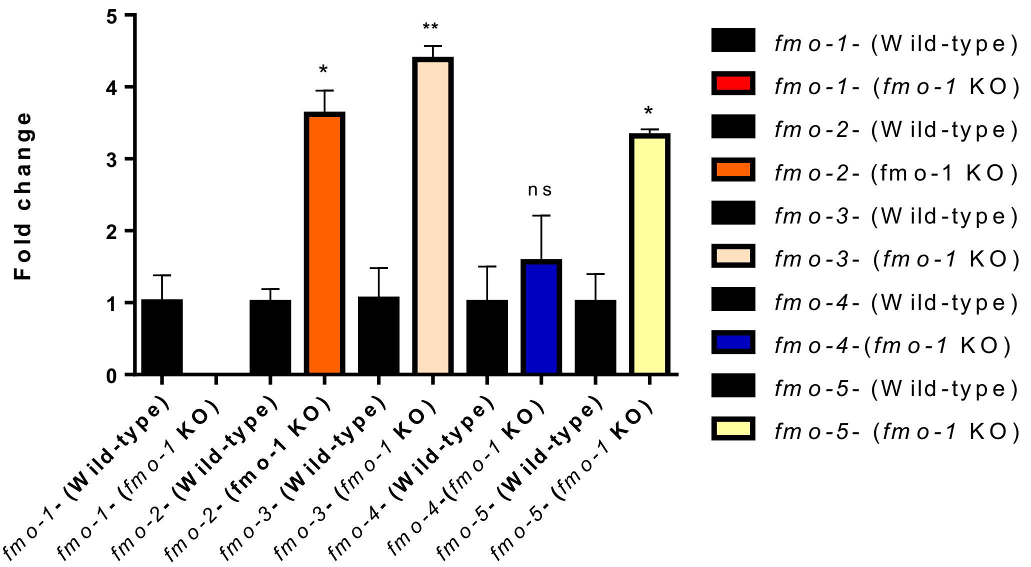

Figure S4B: Transcriptional analysis of the *fmo* genes (*fmo1-5*) in larvae (L2; day 1 post hatching), wild type and *fmo-1* KO *C. elegans* using RT-qPCR. Mean (± SEM) analysed from 3 biological repeats, normalised to the HK gene pmp3 and F35G12.2. HK, Housekeeping genes; KO, knockout; each reaction was run in triplicate. One-way ANOVA: ns, P > 0.05;*, P < 0.05; **, P < 0.01.

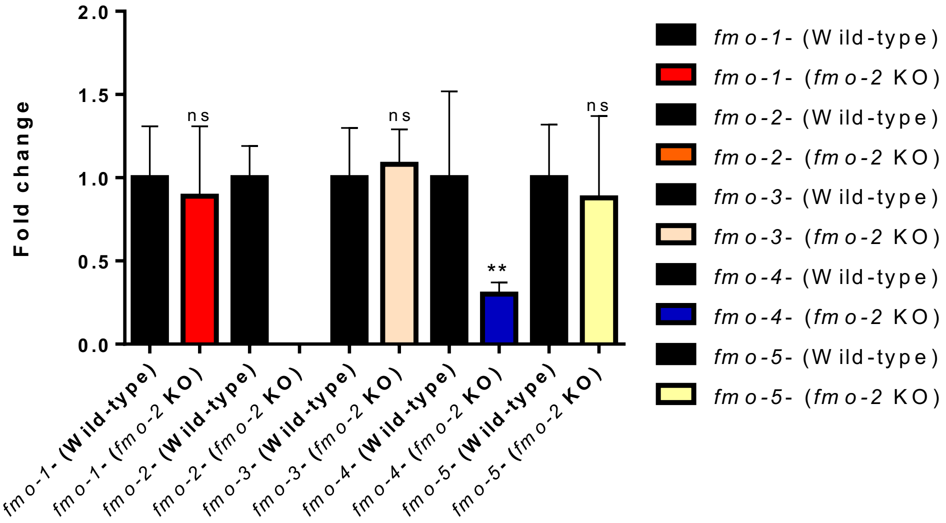

Figure S4C: Transcriptional analysis of the *fmo* genes (*fmo1-5*) in larvae (L2; day 1 post hatching), wild type and *fmo-2* KO *C. elegans* using RT-qPCR. Mean (± SEM) analysed from 3 biological repeats, normalised to the HK gene pmp3 and F35G12.2. HK, Housekeeping genes; KO, knockout; each reaction was run in triplicate. One-way ANOVA: ns, P > 0.05; **, P < 0.01.

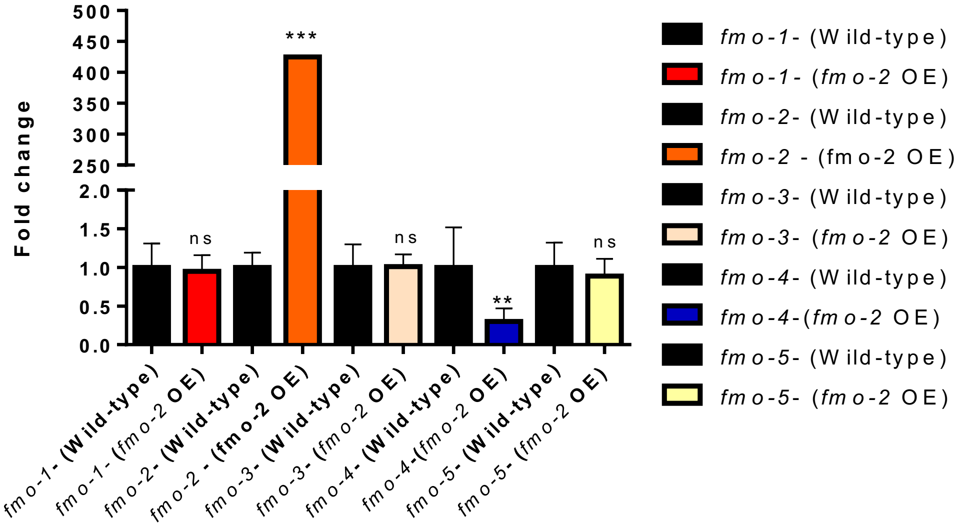

Figure 4D: Transcriptional analysis of the *fmo* genes (*fmo1-5*) in larvae (L2; day 1 post hatching), wild type and *fmo-2* OE *C. elegans* using RT-qPCR. Mean (± SEM) analysed from 3 biological repeats, normalised to the HK gene pmp3 and F35G12.2. HK, Housekeeping genes; KO, knockout; each reaction was run in triplicate. One-way ANOVA: ns, P > 0.05; **, P < 0.01; ***, P < 0.001.

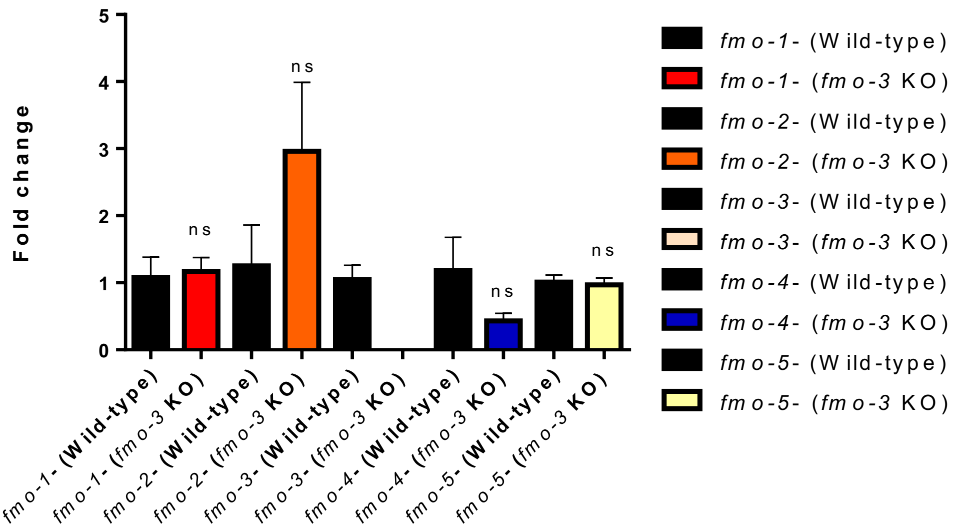

Figure S4E: Transcriptional analysis of the *fmo* genes (*fmo1-5*) in larvae (L2; day 1 post hatching), wild type and *fmo-3* KO *C. elegans* using RT-qPCR. Mean (± SEM) analysed from 3 biological repeats, normalised to the HK gene pmp3 and F35G12.2. HK, Housekeeping genes; KO, knockout; each reaction was run in triplicate. One-way ANOVA: ns, P > 0.05; **, P < 0.01; ***, P < 0.001.

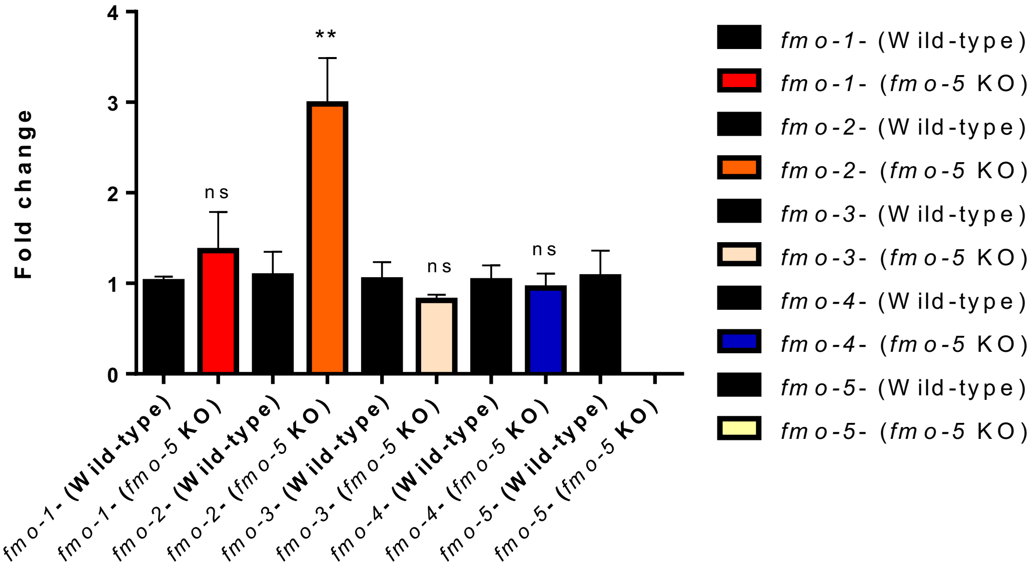

Figure S4F: Transcriptional analysis of the *fmo* genes (*fmo1-5*) in larvae (L2; day 1 post hatching), wild type and *fmo-5* KO *C. elegans* using RT-qPCR. Mean (± SEM) analysed from 3 biological repeats, normalised to the HK gene pmp3 and F35G12.2. HK, Housekeeping genes; KO, knockout; each reaction was run in triplicate. One-way ANOVA: ns, P > 0.05; **, P < 0.01; ***, P < 0.001.

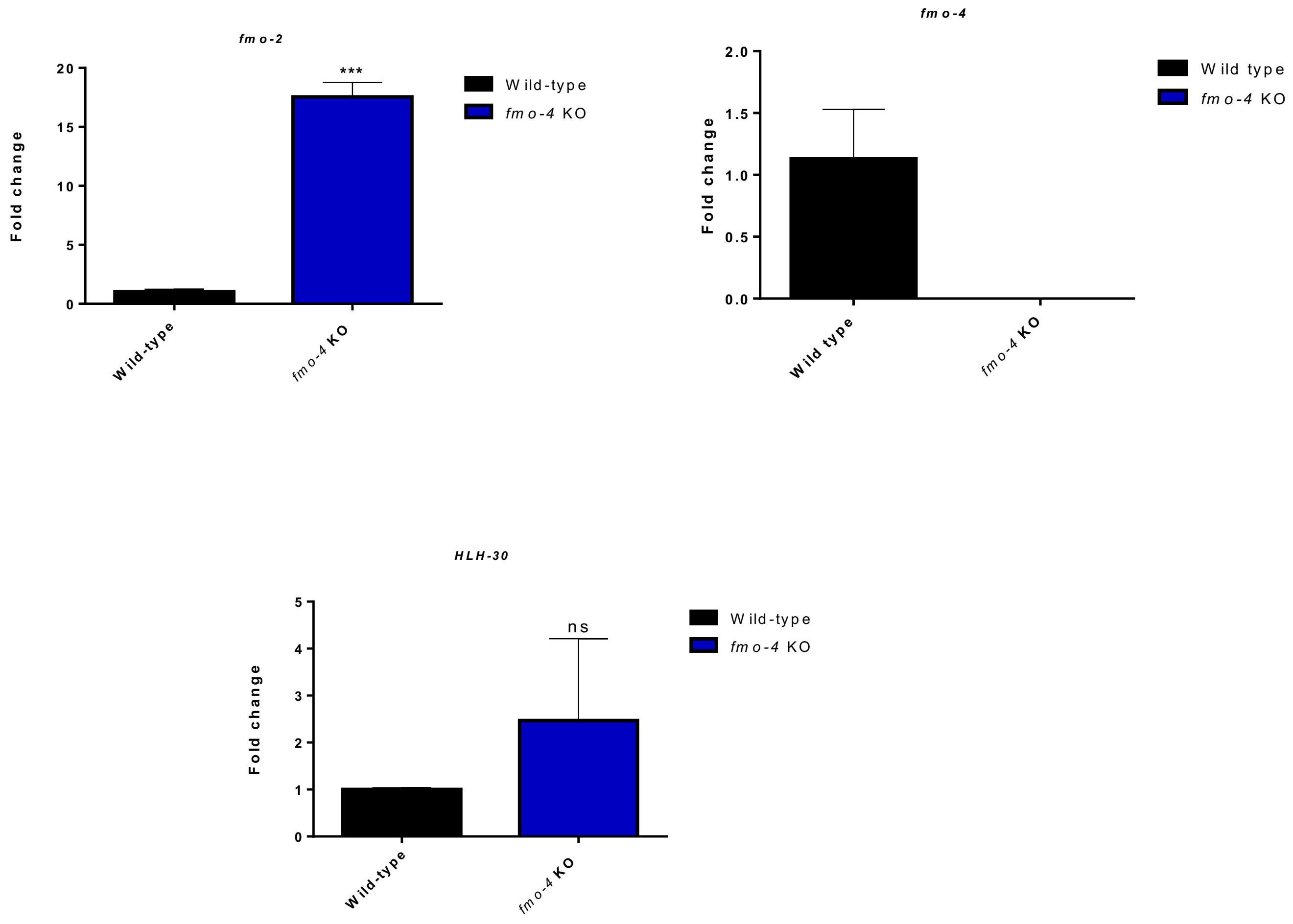

Figure S5: Transcriptional analysis of *fmo*s and *hlh-30* gene in larvae (L2; day 1 post hatching), wild type and *fmo-4* KO *C. elegans* using RT-qPCR. Mean (± SEM) analysed from 3 biological repeats, normalised to the HK gene pmp3 and F35G12.2. HK, Housekeeping genes; KO, knockout; each reaction was run in triplicate. One-way ANOVA: ns, $P > 0.05$; ***, $P < 0.001$

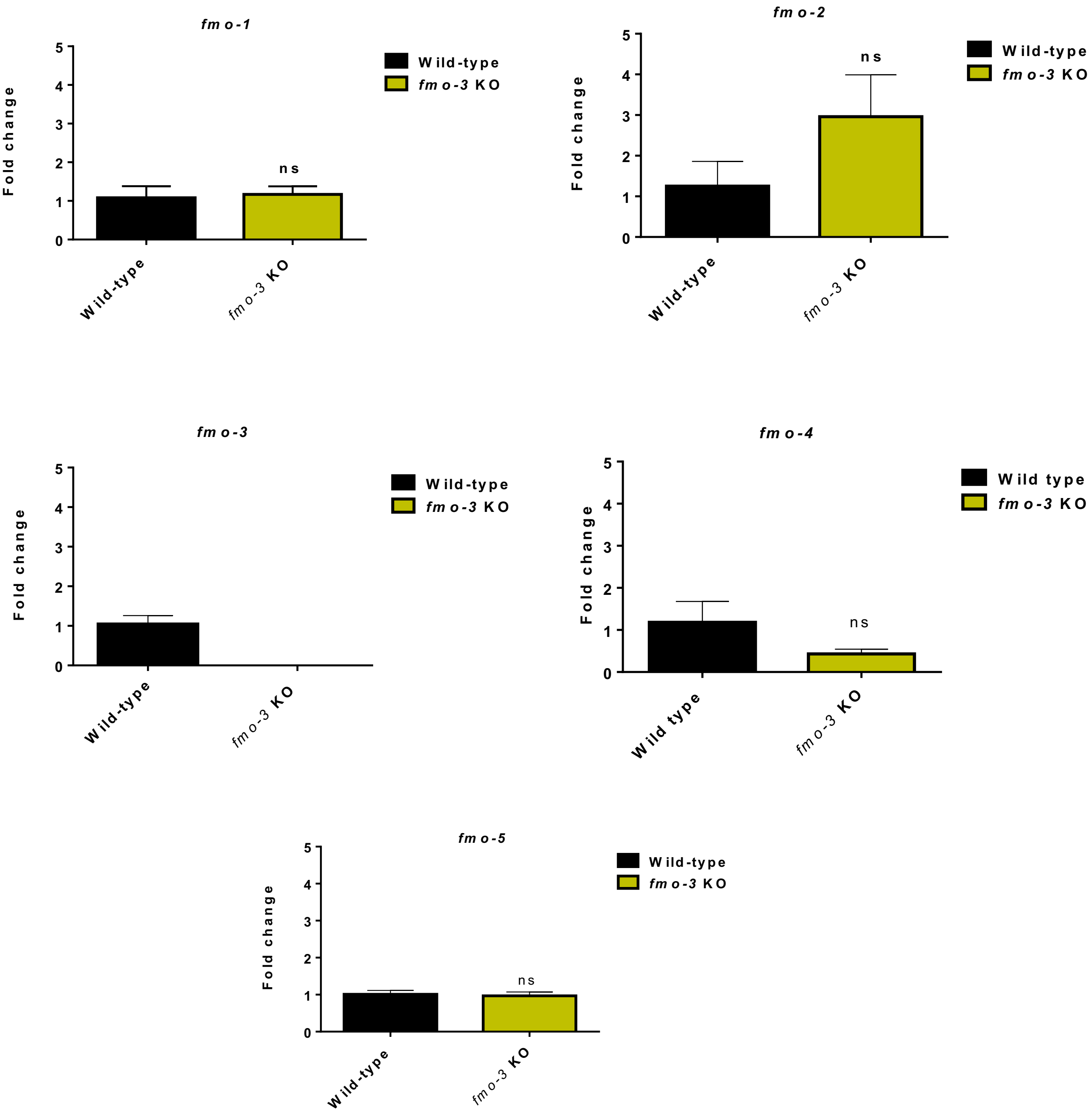

Figure S6: Transcriptional analysis of each *fmo* gene in larvae (L2; day 1 post hatching), wild type and *fmo-3* KO *C. elegans* using RT-qPCR. Mean (± SEM) analysed from 3 biological repeats, normalised to the HK gene pmp3 and F35G12.2. HK, Housekeeping genes; KO, knockout; each reaction was run in triplicate. One-way ANOVA: ns, P > 0.05

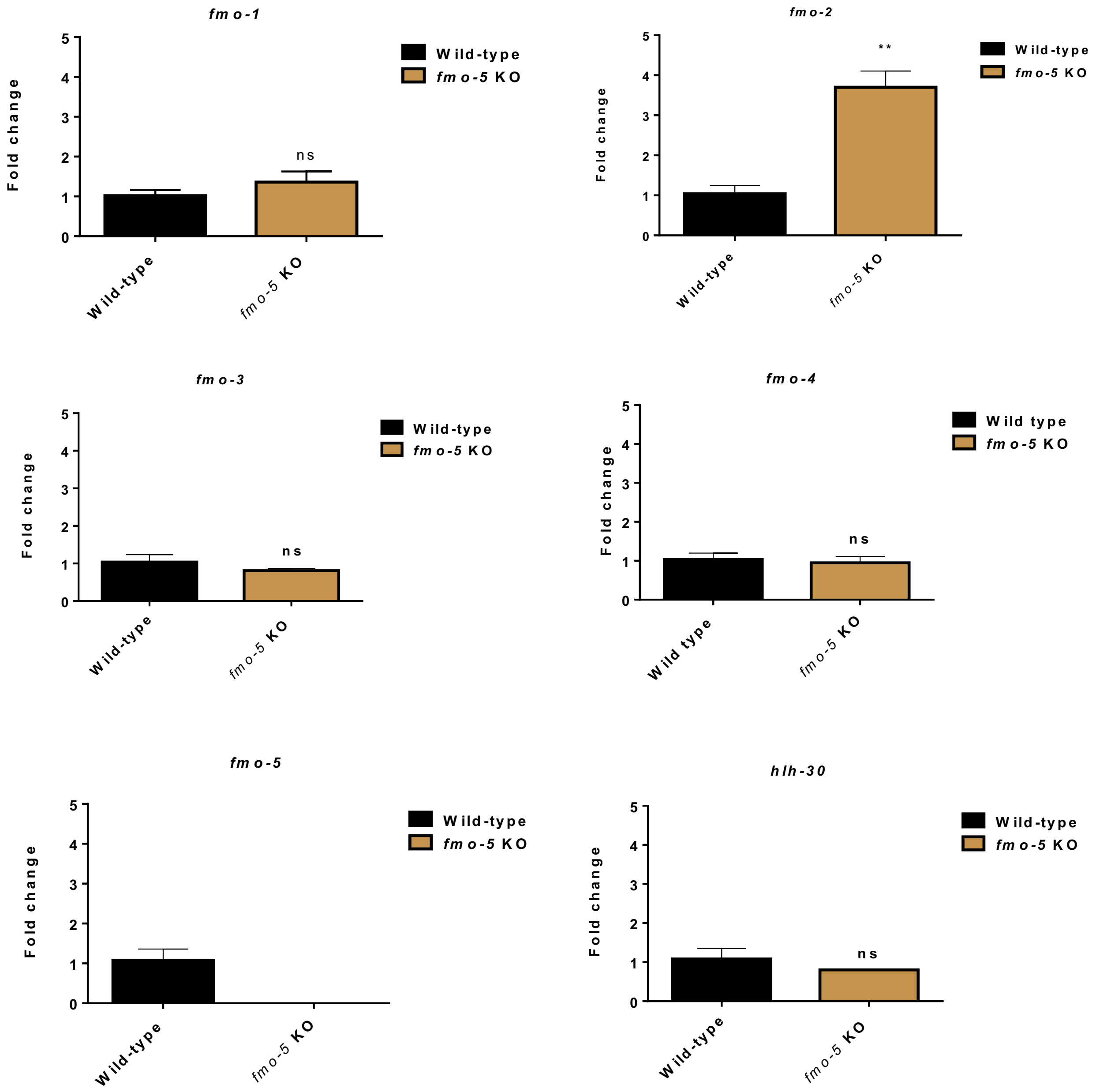

Figure S7: Transcriptional analysis of each *fmo* gene and *hlh-30* in larvae (L2; day 1 post hatching) wild type and *fmo-5* KO *C. elegans* using RT-qPCR. Mean (± SEM) analysed from 3 biological repeats, normalised to the HK gene pmp3 and F35G12.2. HK, Housekeeping genes; KO, knockout; each reaction was run in triplicate. One-way ANOVA: ns, P > 0.05; **, P < 0.01.

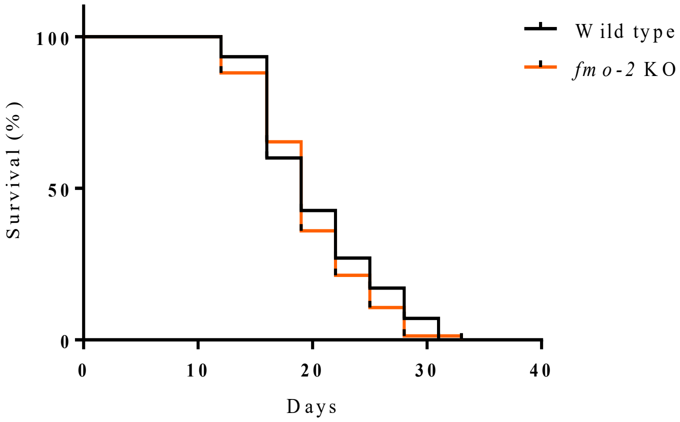

Figure S8. Kaplan-Meier survival curves for wild type and *fmo-2* KO mutants, ns, P > 0.05.

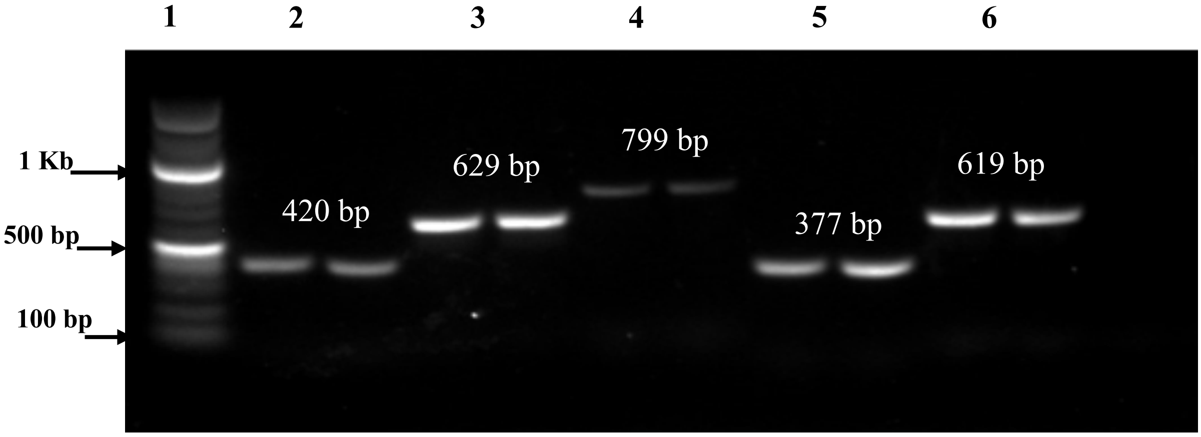

Figure S9. RT-PCR screening of wild type strain with the primers of *C. elegans fmo*s. **1**, 100 bp ladder; **2-6**, Expected product sizes with the primers of the 5 *fmo*s (*fmo-1-5*) respectively. bp, Base pairs.

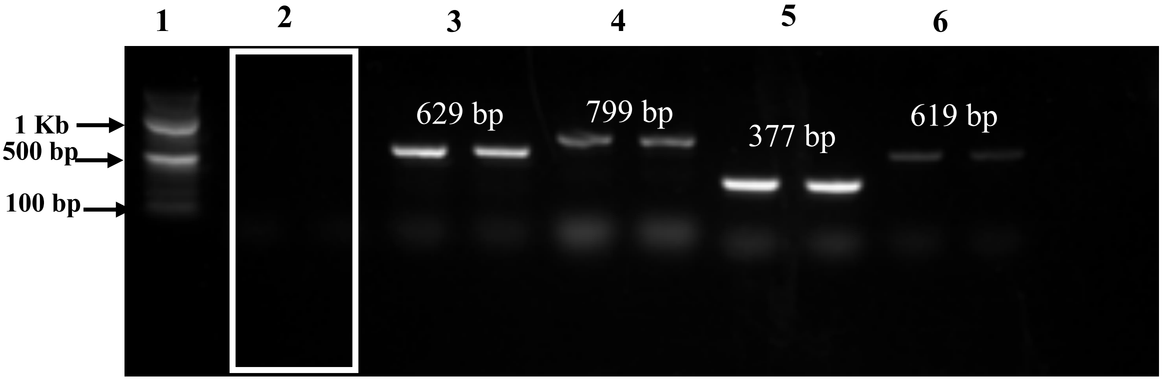

Figure S10. RT-PCR screening of *fmo-1* KO strain with the primers of *C. elegans fmo*s. **1**, 100 bp ladder; **2-6**, Expected product sizes with the primers of the 5 *fmo*s (*fmo-1-5*) respectively. There were no bands with *fmo-1* primers. bp, Base pairs.

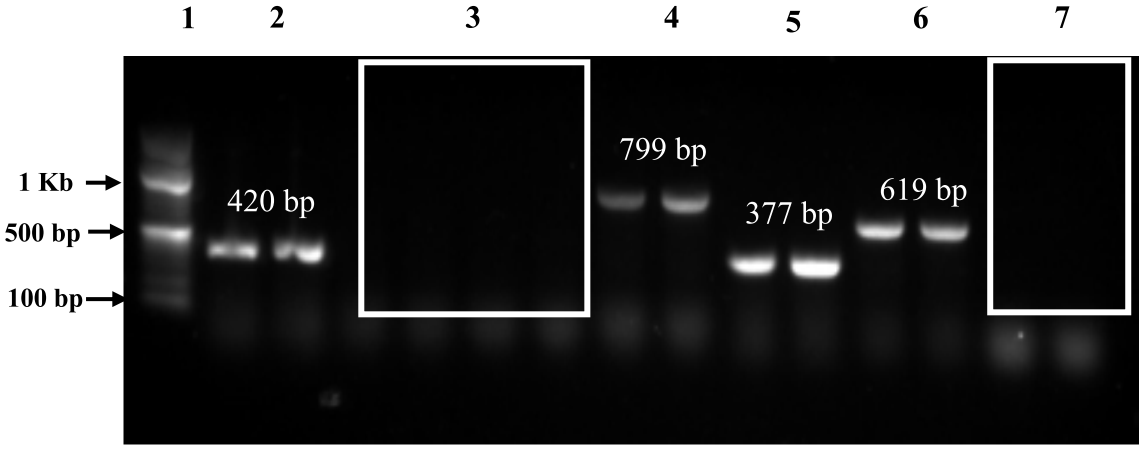

Figure S11. RT-PCR screening of *fmo-2* KO strain with the primers of *C. elegans fmo*s. **1**, 100 bp ladder; **2-6**, Expected product sizes with the primers of the 5 *fmo*s (*fmo-1-5*) respectively; **7**, negative control (it did not contain cDNA. It contained only RNase free water). There were no bands with *fmo-2* primers and with the negative control. bp, Base pairs.

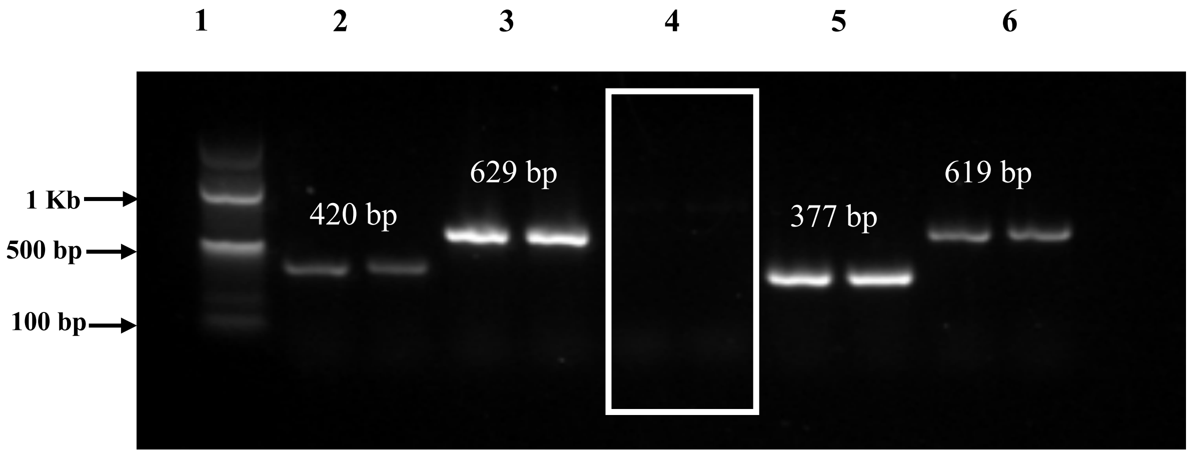

Figure S12. RT-PCR screening of *fmo-3* KO strain with the primers of *C. elegans fmo*s. **1**, 100 bp ladder; **2-6**, Expected product sizes with the primers of the 5 *fmo*s (*fmo-1-5*) respectively.. There were no bands with *fmo-3* primers. bp, Base pairs.

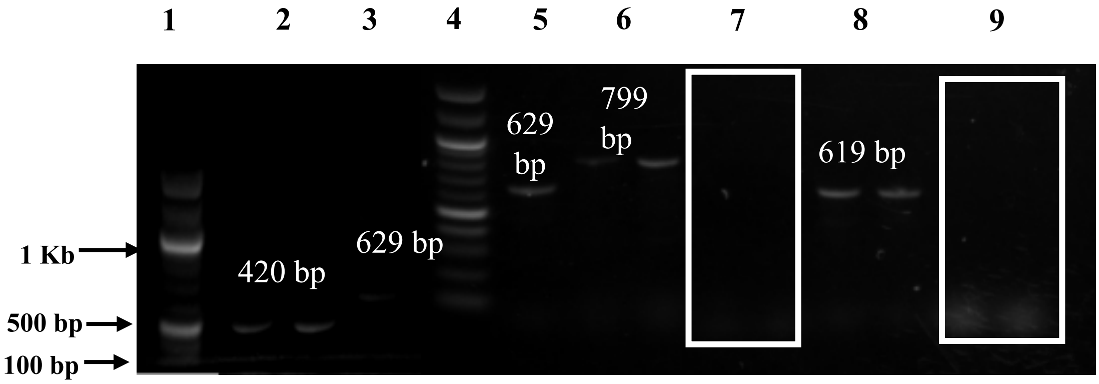

Figure S13. RT-PCR screening of *fmo-4* KO strain with the primers of *C. elegans fmo*s. **1&4**, 100 bp ladder; **2**, *fmo-1* primers; **3&5**, *fmo-2* primers; **6**, *fmo-3* primers; **7**, *fmo-4* primers; **8**, *fmo-5* primers; **9**, negative control. There were no bands with *fmo-4* primers and with the negative control. bp, Base pairs.

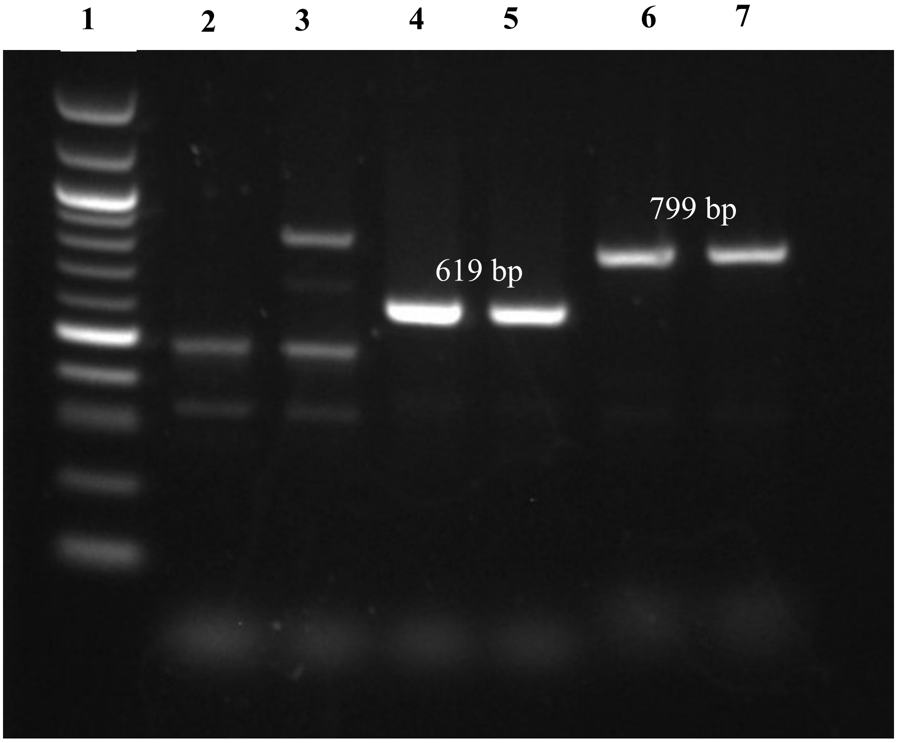

Figure S14: RT-PCR screening of *fmo-5* KO and wild type strains with the primers of *C. elegans fmo-5* and *fmo-3*. 1, 100 bp ladder; 2&3 *fmo-5* KO cDNA with *fmo-5* primers; 4&5, wild type cDNA with *fmo-5* primers; 6, *fmo-5* KO cDNA with *fmo-3* primers; 7, wild type cDNA with *fmo-3* primers. There were no bands with *fmo-5* primers with the cDNA of *fmo-5* KO at the correct band size (619 bp), but there is a band at 456 bp as the mutant is achieved by deletion of 163 bp in exon. bp, Base pairs.

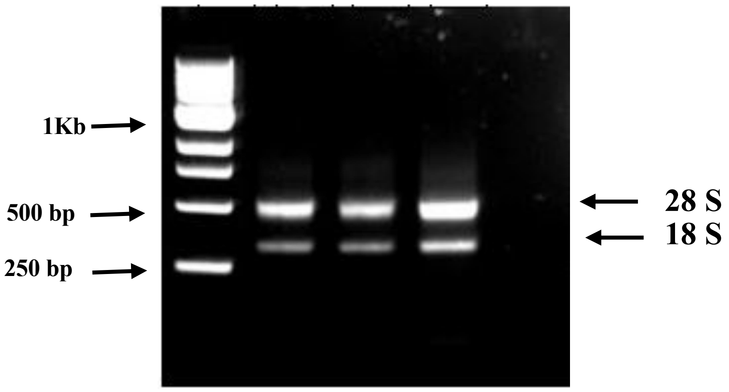

Figure S15. Gel electrophoresis of the yielded RNA of *C. elegans fmo-2* KO larvae. **1**, 1Kb ladder; **2**, **3** and **4**, three biological repeats of *fmo-2* KO RNA. Two clear bands at 18S and 28S.

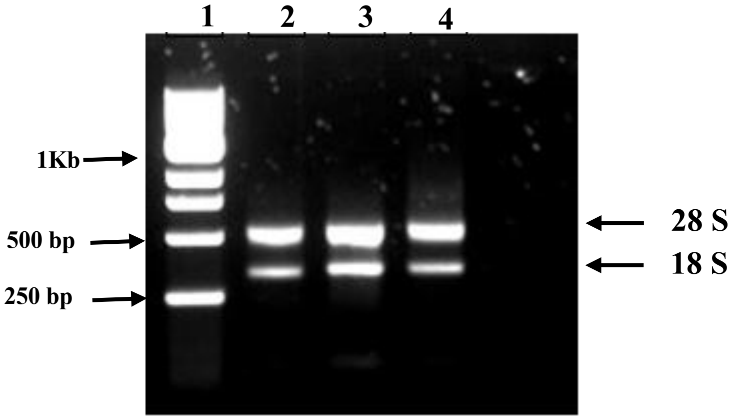

Figure S16. Gel electrophoresis of the yielded RNA of *C. elegans fmo-2* OE larvae. **1**, 1Kb ladder; **2**, **3** and **4**, three biological repeats of *fmo-2* OE RNA. Two clear bands at 18S and 28S.

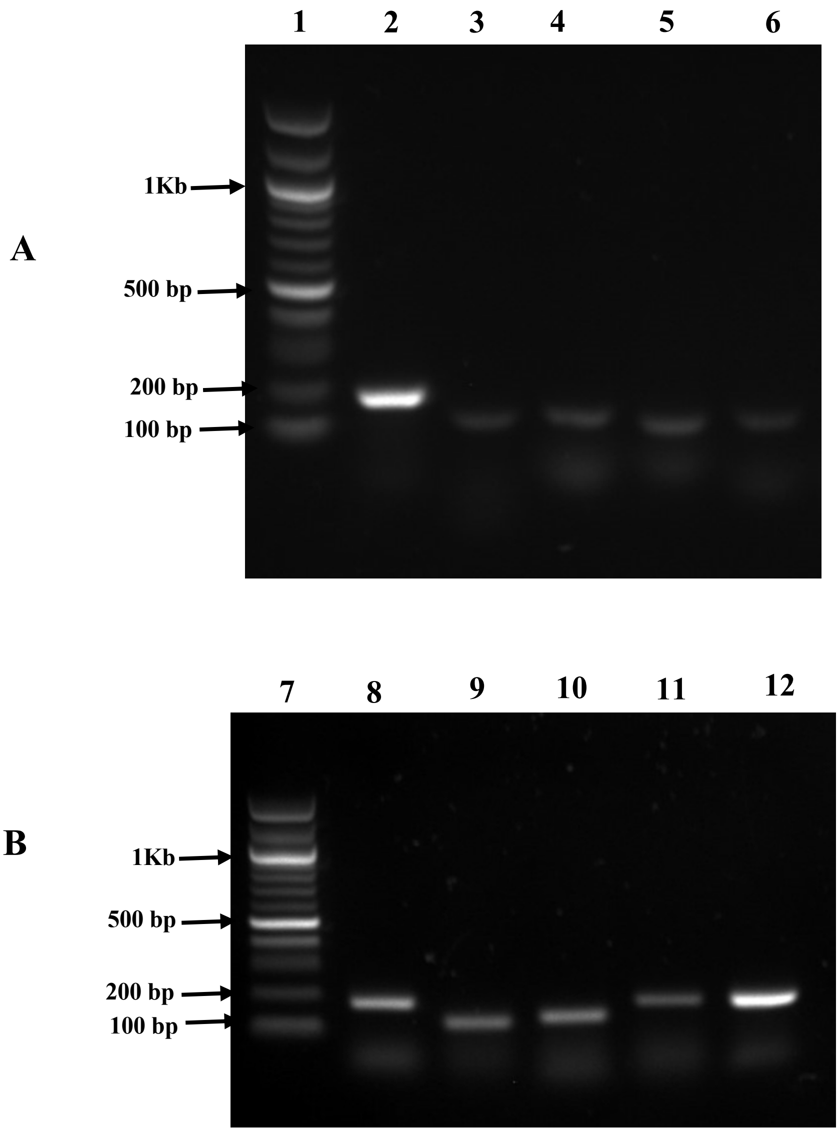

Figure S17. Validation of qPCR primers genes: gel electrophoresis of qPCR primer testing PCR. **A**, housekeeping genes qPCR primers; **B**, *C. elegans* five *fmo* genes (*fmo1-5*). PCR of derived wild type *C. elegans* cDNA was preformed, using qPCR cycling parameters with annealing temperature set to 60 °C, for all qPCR primers, to test for gDNA contamination and if secondary structures were formed. The PCR were resolved on 1% agarose gel, expected amplicon sizes were observed with no secondary structures or gDNA contamination present. **1&7**, 100 bp ladder; **2,** *tba-1* primers; **3**, *pmp-3* primers; **4,** *rbd-1* primers; **5,** Y45F10D.4 primers; **6,** F35G12.2 primers; **8,** *fmo-1* primers; **9,** *fmo-2* primers, **10,** *fmo-3* primers, **11**, *fmo-4* primers, **12**, *fmo-5* primers. bp, Base pair.

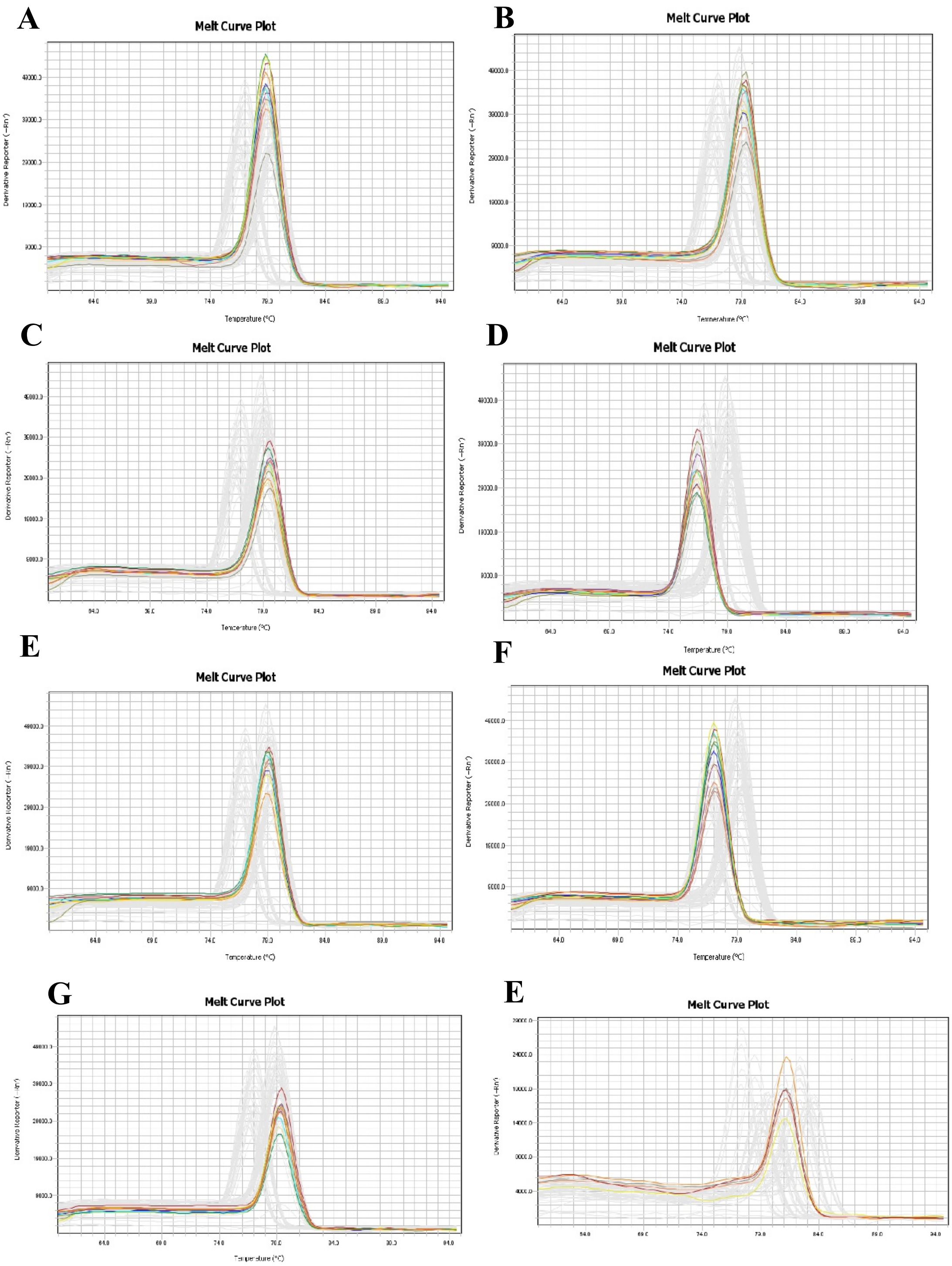

Figure S18. Validation of qPCR primers: Melt curve analysis of qPCR primers. Melt curves were calculated and produced in QuantStudio Design & Analysis Software during all qPCR runs. The above curves were produced during the initial primer validation with 10-fold serial dilutions of wild type, embryo, larvae and adult cDNA. Curves were checked to ensure single peaks were produced, indicative of single target amplification from template. If secondary products were formed, multiple non uniform peaks would be produced in the graph, which

was not the case. **A**, *pmp-3* primers; **B**, F35G12.2 primers; **C**, *fmo-1* gene primers; **D**, *fmo-2* primers; **E**, *fmo-3* primers; **F**, *fmo-4* primers; **G**, *fmo-5* primers; **H**, *hlh-30* primers.

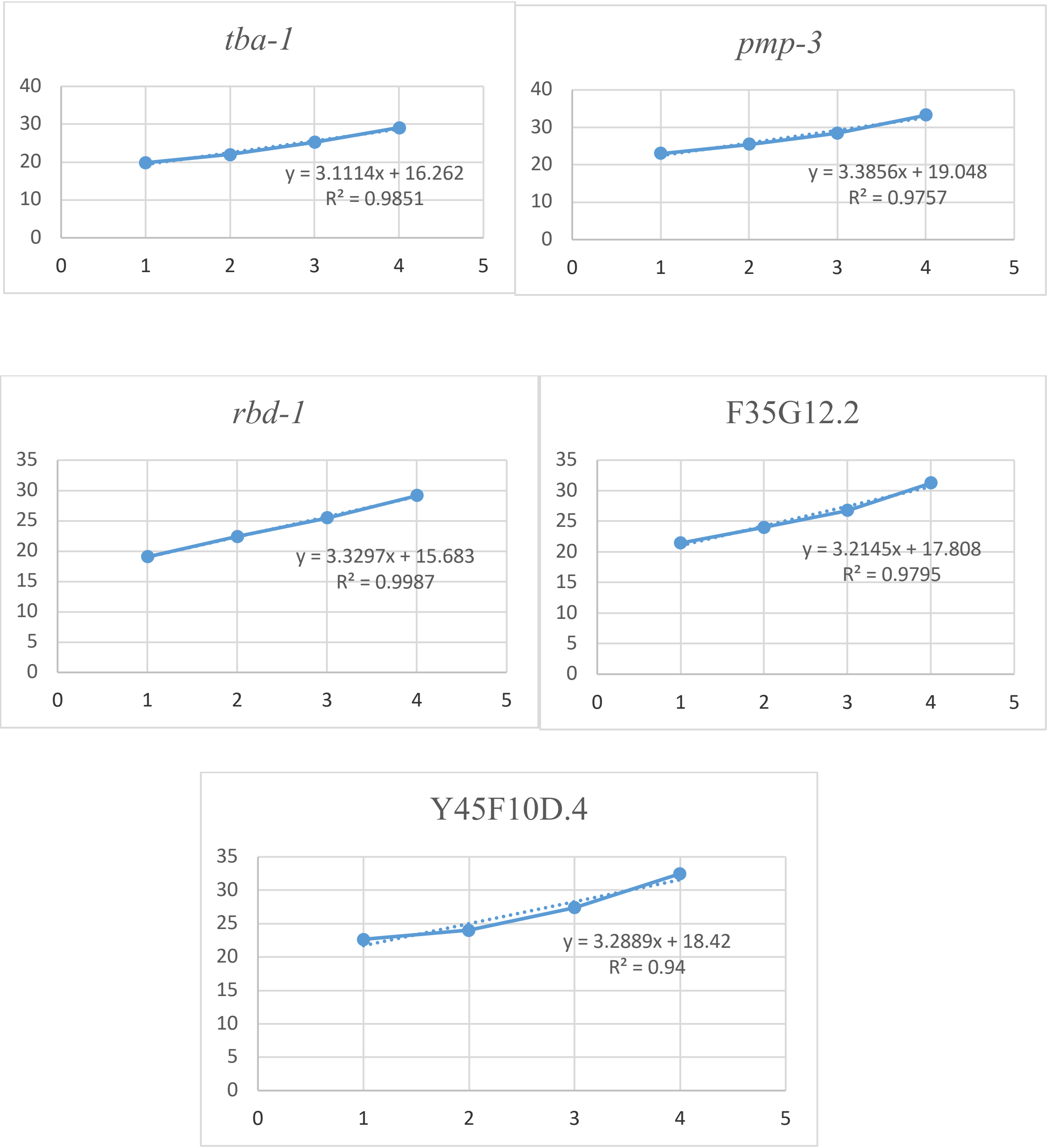

Figure S19. Validation of qPCR primers of housekeeping genes. Amplification efficiencies were estimated via PCR using 10-fold dilution of wild type adults. Standard curves were produced, equation of the straight line determined, and slope used to estimate primer efficiency.

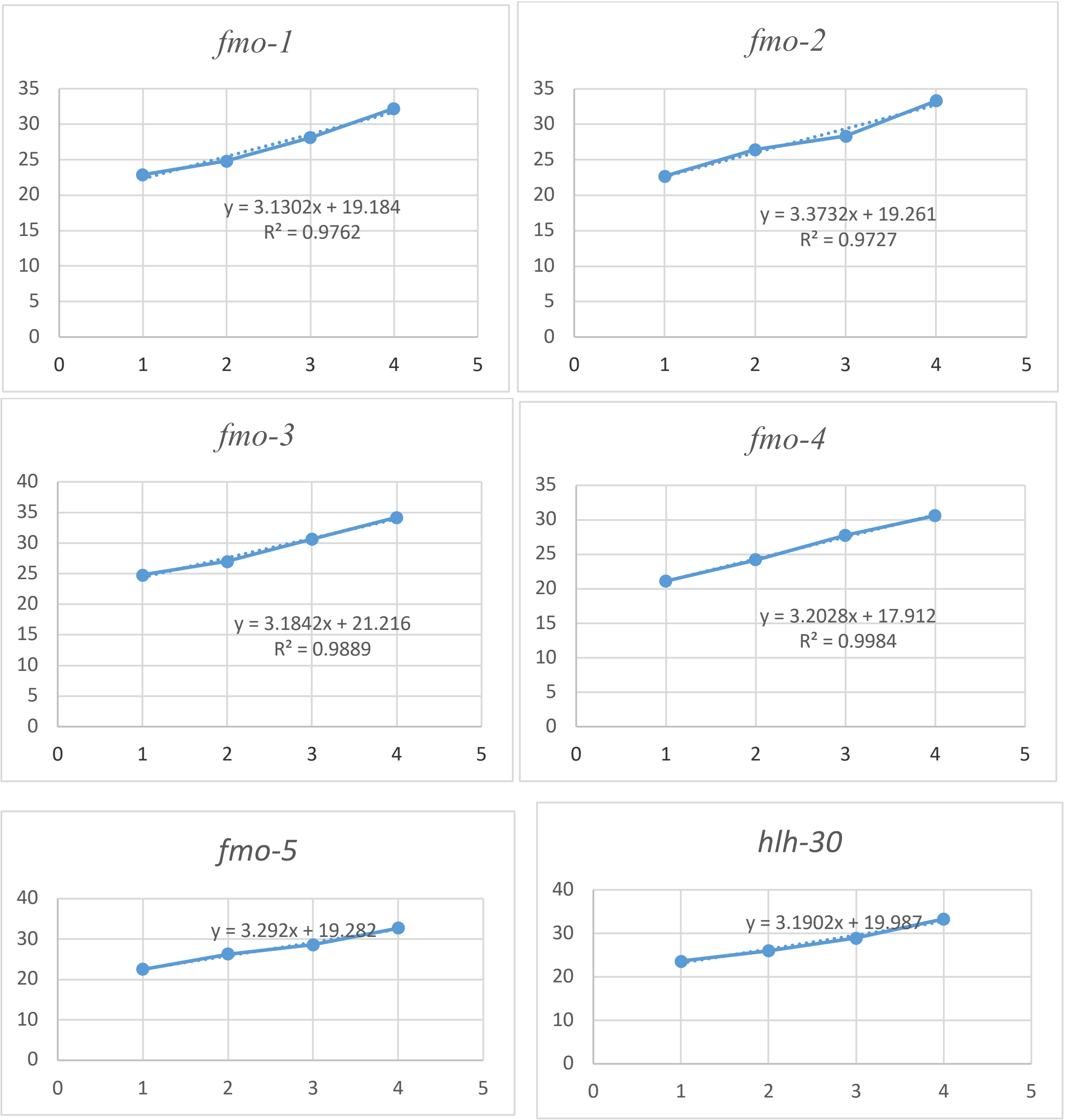

Figure S20. Validation of qPCR primers of *C. elegans* five *fmo* (*fmo1-5*) genes and *hlh-30* gene. Amplification efficiencies were estimated via PCR using 10-fold dilution of wild type adults. Standard curves were produced, equation of the straight line determined, and slope used to estimate primer efficiency.

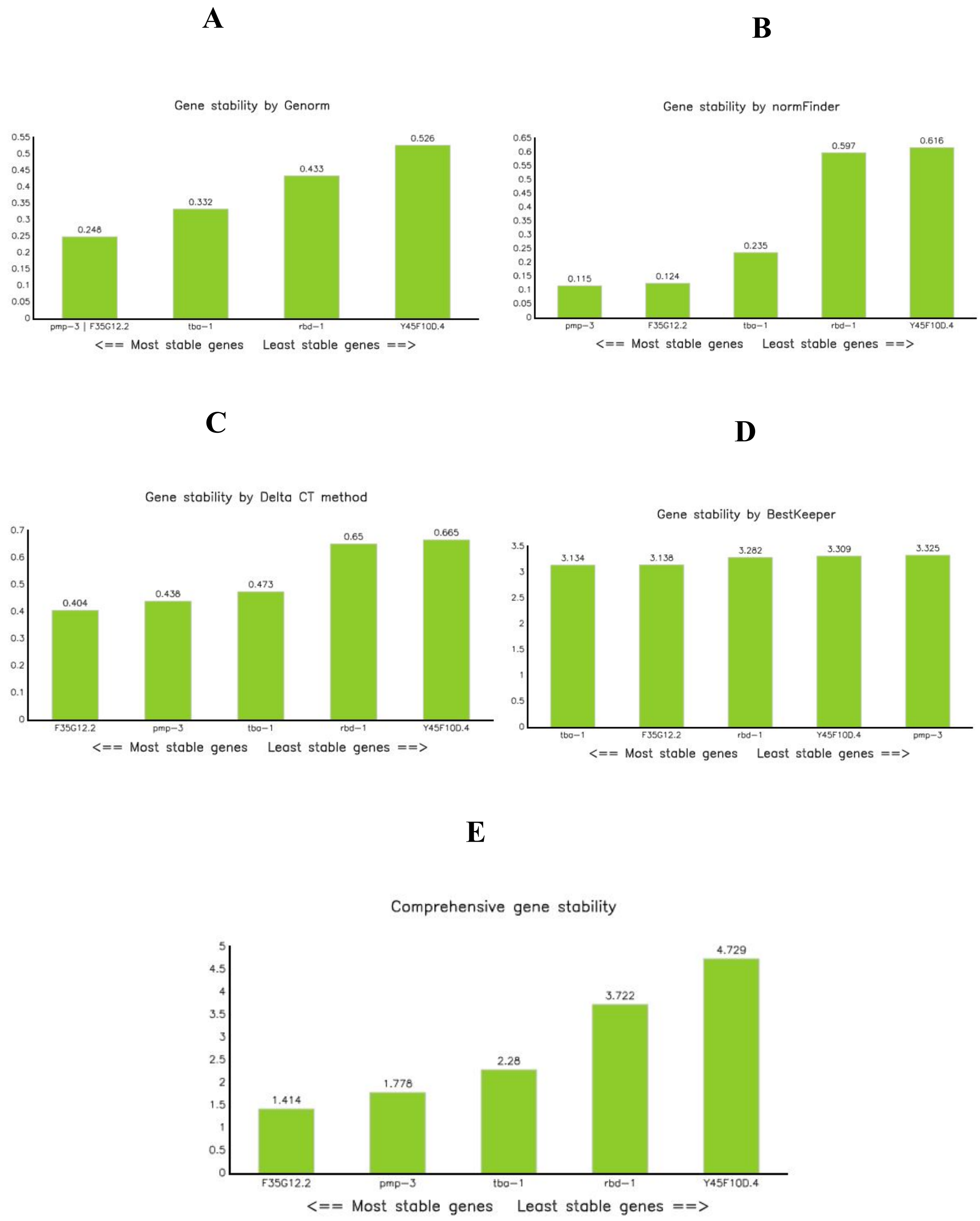

Figure S21. The average expressing stability of the candidate housekeeping genes using the four common algorithm. **A,** geNorm algorithm; **B,** normFinder algorithm; **C,** delta *Ct* algorithm; **D,** BestKeeper algorithm; **E,** comprehensive gene stability ranking.

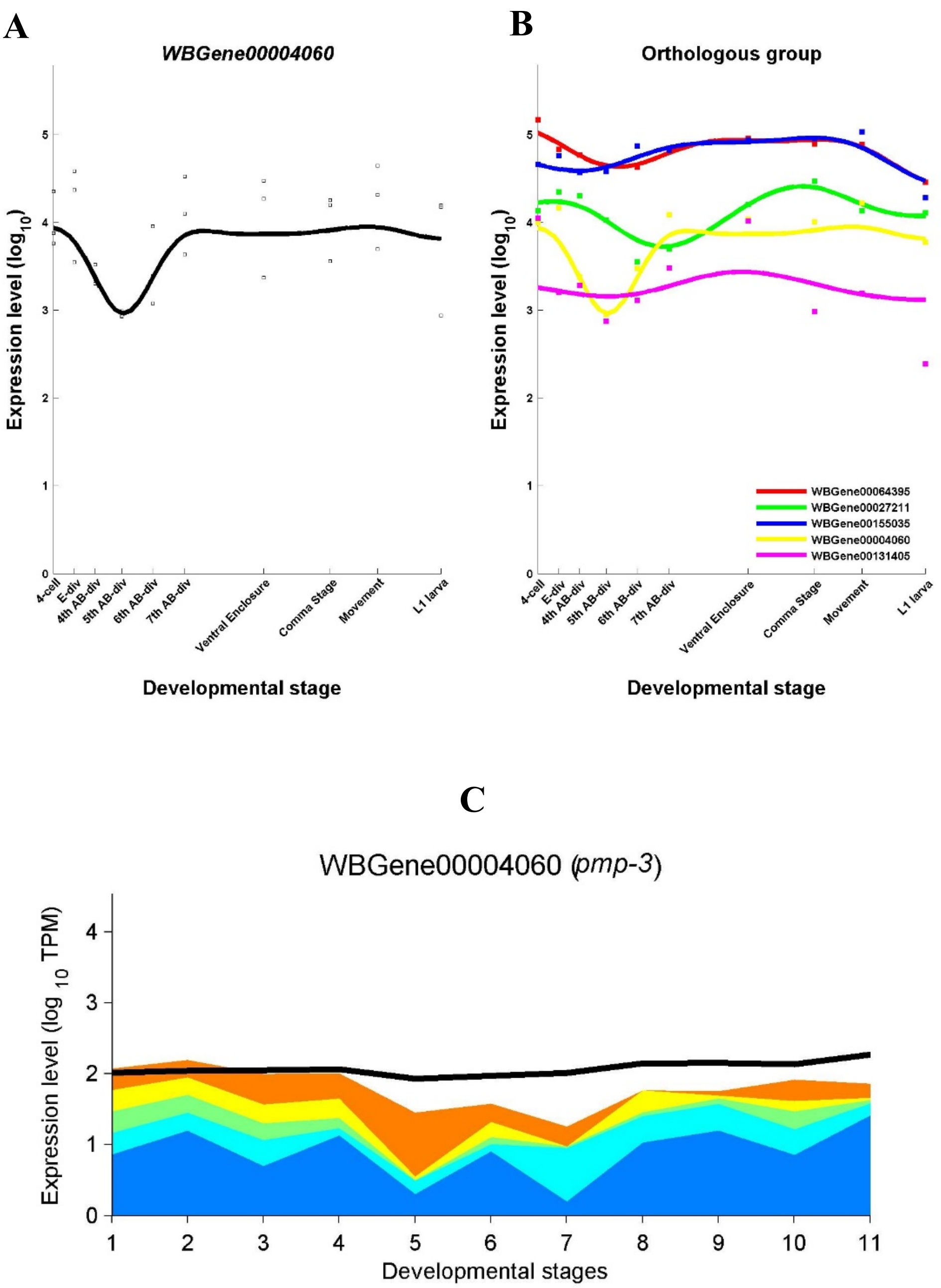

Figure S22. A, Embryonic gene expression profile of *pmp-3* in *C. elegans*. **B**, Comparative gene expression profiles for the orthologous group; blue, *C. brenneri*; green, *C. briggsae*; red, *C. remanei*; yellow, *C. elegans*; pink, *C. japonica.* **C***,* showing gene expression for the *pmp-3* gene; where the black line represents expression in the whole embryo, and the coloured areas correspond to the blastomere lineage (same colours as in the lower panel). The transcriptome of each sample was determined using CEL-Seq (reproduced from Hashimshony *et al.,* 2015). Images were obtained also from wormbase. These figures are showing the transcriptional stability of *pmp-3* throughout the development stages and relevant for our study as we are comparing three stages including; embryo, Larvae and Adult stage.

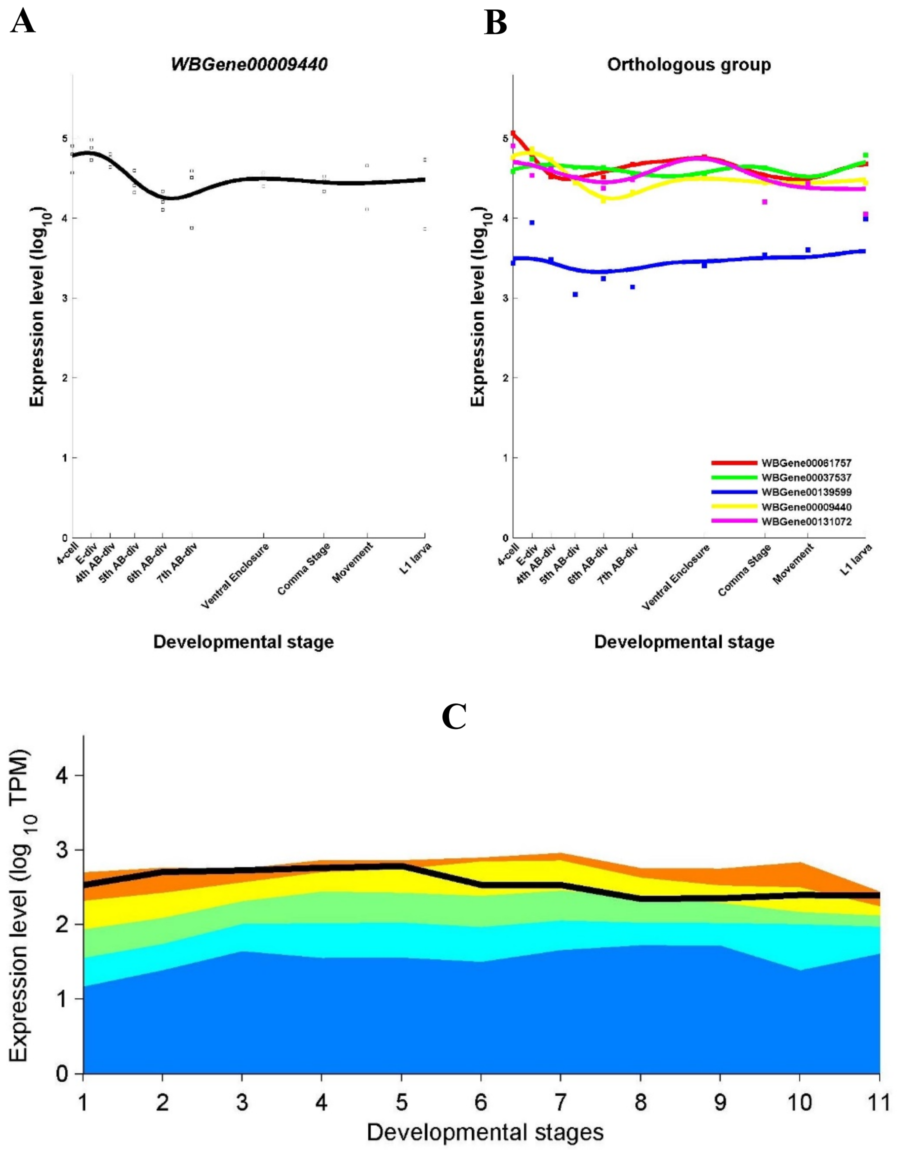

Figure S23. A, Embryonic gene expression profile of *F35G12.2* in *C. elegans*. **B**, Comparative gene expression profiles for the orthologous group; blue, *C. brenneri*; green, *C. briggsae*; red, *C. remanei*; yellow, *C. elegans*; pink, *C. japonica.* **C***,* showing gene expression for the *F35G12.2* gene; where the black line represents expression in the whole embryo, and the coloured areas correspond to the blastomere lineage (same colours as in the lower panel). The transcriptome of each sample was determined using CEL-Seq (reproduced from Hashimshony *et al.,* 2015). Images were obtained also from Wormbase (https://wormbase.org/). These Figures are showing the transcriptional stability of *F35G12.2* throughout the development stages.

Table S1. BLASTp analysis of *C. elegans* FMO-4 against the rest of *C. elegans* FMOs (FMO1-3 and -5).

Organism	Protein	Total Score	Maximum Score	Length (amino acid)	Query cover (%)	Identity (%)	E-value	Accession NCBI
C. elegans	FMO-1	419	419	533	84	41.19	9e-146	Query_53913
	FMO-2	423	423	529	84	42.94	3e-147	Query_42587
	FMO-3	398	398	512	86	41.19	9e-138	Query_12493
	FMO-5	421	421	518	86	42.97	1e-146	Query_33525

All information was provided by (BLASTp, NCBI)

Table S2. RNA yield and purity of each biological repeat of different lifecycle stages of wild type *C. elegans* and larvae stages of *fmo-1* KO, *fmo-2* KO, *fmo-4* KO and *fmo-2* OE.

Stage	Yield ng/µl	Purity A260/A280
Embryo	290	2.07
Embryo	165	2.03
Embryo	398	2.04
Larvae 1	413	2.02
Larvae 1	294	2.02
Larvae 1	448	2.05
Adult	1448	2.01
Adult	1697	1.93
Adult	1759	1.92
fmo-1 KO larvae	295	2.05
fmo-1 KO larvae	285	2.04
fmo-1 KO larvae	597	2.05
fmo-4 KO larvae	131	2.04
fmo-4 KO larvae	465	2.06
fmo-4 KO larvae	339	2.04
fmo-2 KO larvae	708	2.01
fmo-2 KO larvae	504	2.03
fmo-2 KO larvae	932	2.03
fmo-2 OE larvae	502	2.02
fmo-2 OE larvae	1080	2.01
fmo-2 OE larvae	1059	2.00
fmo-5 KO larvae	1446	1.97
fmo-5 KO larvae	240	2.032
fmo-5 KO larvae	1110	2.012

Table S3. Designed primers of the candidate HK genes with the expected product size for RT-qPCR.

**Gene**	**Gene description**	**Forward primer**	**Reverse primer**	**Product size (bp)**
tba-1	TuBulin, alpha family member	TCAACACTGCCAT CGCCGCC	TCCAAGCGAGACCAGG CTTCAG	193
pmp-3	Peroxisomal membrane protein related	TGGCCGGATGATG GTGTCGC	ACGAACAATGCCAAAG GCCAGC	114
F35G 12.2	Hypothetical protein	ACTGCGTTCATCC GTGCCGC	TGCGGTCCTCGAGCTC CTTC	147
Y45F 10D.4	Putative iron-sulphur cluster assembly enzyme	CGAGAACCCGCGA AATGTCGGA	CGGTTGCCAGGGAAGA TGAGGC	100
rbd-1	RBD (RNA binding domain) protein	GGTCAGATTTCCG ATGCGTCGCT	ACTTGCTCCAGGCTCT CGGC	130

bp, Base pairs.

Table S4. Designed primers of the five *C. elegans fmo* genes and with the expected product size for RT-qPCR.

Gene	Forward primer	Reverse primer	Product size (bp)
fmo-1	TTGTGATGTCTACTGGATTCTC	GCATTTCAGATAGTGGCATAA	186
fmo-2	GGTCTTCGCCTCTTCTTT	GGTTGTAGTTGCCATTCG	126
fmo-3	ACGACGAATTACCAAATAGAA	AATCCAGTTGCTAGAATCAC	139
fmo-4	GCGGAGATTATTGGATGTT	TTCAGTGGCTTCTTGACT	182
fmo-5	AGCCTTTATGATCCTTATAGTCA	GACGGAGTTATGATCTGCT	121
hlh-30	ACATGAAACTCAACAAAGGA	CGATTCCAGCGATTTCTG	116

bp, Base pairs.

Table S5. Slope and efficiency (E) of each gene of the five housekeeping genes and the five *C. elegans fmo* (*fmo1-5*) genes.

Gene	Slope	Efficiency	E % (90-110)
tba-1	3.11	2.10	109
pmp-3	3.39	1.97	97.4
rbd-1	3.33	2.00	99.7
F35G12.2	3.21	2.05	104
Y45F10D.4	3.29	2.01	101
fmo-1	3.13	2.09	108
fmo-2	3.37	1.98	97.9
fmo-3	3.18	2.06	106
fmo-4	3.20	2.05	105
fmo-5	3.50	1.93	93.1
fmo-5	3.292	2.01	101
hlh-30	3.19	2.05	105

Table S6. Primer design of each *fmo* gene and the expected product sizes from RT-PCR reaction of wild type *C. elegans* cDNA.

Gene	Forward	Reverse	product size (bp)
fmo-1	TCACGAATACAGTTGTCGAA	ATACTCTCGCTTGCATTTCA	420
fmo-2	CTTCTGGATTACCGTCGATT	TGGTATAAGCCAAGTTCCAC	629
fmo-3	TGAAGTCGGAGGATTATGGA	TCCTGGTTTTACTCGAACTG	799
fmo-4	CAAAAGTTACGCAGAGCATT	GACTGACACGGTTGAAAATC	377
fmo-5	AGGACTGTGGAACTTTAAGC	ATCAACTAGCTGCATTTGGA	619

bp, Base pairs.
